# Supplementary material for: Targeted Blood Plasma Proteomics and Hemostasis Assessment of Post COVID-19 Patients with Acute Myocardial Infarction
Source: Int J Mol Sci. 2023 Mar 30;24(7):6523. doi: 10.3390/ijms24076523 (PMC10094800; doi:10.3390/ijms24076523)
Supplement: Supplementary file 1 [file ijms-24-06523-s001.zip › Supplementary Materials File S1.pdf]

Supplementary Materials

Materials and Methods (Extended)

Blood sampling

For blood sampling we used the S-Monovette blood collection system (Sarstedt AG & Co.KG, Germany). We used S-Monovette 2,7 ml K3E (REF 05.1167.001) for complete blood count; S-Monovette 7,5 ml Z-Gel (REF 01.1602.001) for biochemical blood tests; and S-Monovette 5 ml 9NC (REF 05.10I 71.001) for coagulation blood tests.

For ROTEM, thrombodynamics and aggregometry we used S-Monovette 5 ml 9NC (REF 05.10I 71.001). For ROTEM and aggregometry we used whole blood, and for thrombodynamics in both modes we used platelet free plasma obtained by two consecutive centrifugations (1600g x 15 min, RT, then plasma transfer to a new 1,5 ml tube following by 1000g x 5 min, RT).

Plasma used in proteomics study was obtained from whole blood in S-Monovette 2,7 ml K3E (REF 05.1167.001) by centrifugation 4000g x 10 min, RT and then aliquoted and frozen at -80 °C.

Laboratory and instrumental analysis

A complete blood count (hemoglobin level, red blood cells (RBC), white blood cells (WBC), platelet count, etc.) was performed on Siemens ADVIA 2120i Hematology System with Autoslide (Siemens Healthcare Diagnostics Inc, USA).

Biochemical blood tests (serum creatinine, alanine aminotransferase (ALT), aspartate aminotransferase (AST), etc.) were performed on Siemens ADVIA 2400 Chemistry Analyzer (Siemens Healthcare Diagnostics Inc, USA). Serum creatinine was measured with ADVIA® Chemistry CREA_2 Creatinine Reagents (REF 3039070); ALT was measured with ADVIA® Chemistry ALT (GPT) Reagents (REF 7501976); AST was measured with ADVIA® Chemistry AST(GOT) Reagents (REF 7499718). All reagents for biochemical blood test were from the ADVIA® trade mark (Siemens Healthcare Diagnostics Inc, USA).

Coagulation blood tests (D-dimer, activated partial thromboplastin time (APTT), prothrombin time (PT), international normalized ratio (INR), antithrombin III (AT-III), Quick prothrombin time test (PT Quick) were performed on ACL TOP 300 CTS (Instrumentation Laboratory, USA). D-dimer was measured with HemosIL D-Dimer HS 500 (REF 00020500100); APTT was measured with HemosIL SynthASil (REF 00020006800); PT was measured with HemosIL RecombiPlasTin 2G (REF 00020003050); AT-III was measured with HemosIL Liquid Antithrombin (REF 00020300400); INR and PT Quick were calculated on ACL TOP 300 CTS (Instrumentation Laboratory, USA). All reagents for coagulation blood tests were from the HemosIL trade mark (Instrumentation Laboratory, USA).

Rotational thromboelastometry

Rotational thromboelastometry was performed in NATEM mode on the ROTEM (The Tem Innovations, GmbH, Germany). For measurement in NATEM mode we used ROTEM® star-tem® 20 (REF 503-10-US) (The Tem Innovations, GmbH, Germany).

Thrombodynamics

Thrombodynamics was conducted on a Thrombodynamics Analyzer System T-2 (HemaCore LLC, Russia) according to the standard technique. For the thrombodynamic test mode we used

the Thrombodynamics kit (REF K2-02-10) and for the fibrinolysis test mode we used the Thrombodynamics TDL kit (REF TDL-10). All reagents for thrombodynamic and fibrinolysis tests were from the HemaCore trade mark (HemaCore LLC,Russia and HemaCore SA, Switzerland).

Impedance Aggregometry

A Multiplate® analyzer (Roche Diagnostics International Ltd, Rotkreuz, Switzerland) was used to assess impedance aggregometry. For all tests we used Test Cells (REF 06675590); for arachidonic acid (ASPI) platelet activation we used ASPItest reagent (REF 08847533190), for adenosine diphosphate (ADP) platelet activation we used ADPtest reagent (REF 08847550190), and for thrombin receptor-activated peptide-6 (TRAP-6) platelet activation we used TRAPtest reagent (REF 08847509190). All reagents for impedance aggregometry were the Multiplate® analysis trade mark (Roche Diagnostics International Ltd, Switzerland).

Targeted blood plasma proteomics

Targeted proteomic analysis was carried out using liquid chromatography-tandem mass spectrometry (LC-MS/MS) with multiple reaction monitoring (MRM). Synthetic stable-isotope labeled internal standard (SIS) and natural (NAT) synthetic proteotypic peptides were used for measuring the corresponding proteins in plasma. The selected 227 SIS and NAT synthetic peptides had been previously validated for use in LC/MRM-MS experiments for blood plasma [12]. The SIS peptide mixture was spiked in each sample at a balanced concentration which was optimized in previous experiments with a dilution series of samples. Standard curves were generated using NAT and SIS peptide standards with bovine serum albumin (BSA) as a surrogate matrix as previously described in detail [11].

Sample preparation was carried out using 10 μL of plasma. Before trypsinolysis, the samples were reduced with 5 mM dithiothreitol (30 min, +37 °C) and alkylated in the dark with 20 mM iodoacetamide (30 min). TPCK Treated trypsin (Worthington, USA) was added in an enzyme:protein ratio of 1:25, hydrolysis was performed at +37°C overnight. The reaction was quenched by adding formic acid (FA) up to 0.5%. The SIS peptide mixture was spiked in each sample followed by desalting by solid-phase extraction using plates (Oasis HLB 96-well Microelution Plate, Waters, USA). The eluate was lyophilized and dissolved in 0.1% FA to a concentration of ~1 mg/mL for further LC-MS/MS analysis.

All samples were analyzed in duplicate by high performance liquid chromatography mass spectrometry (HPLC-MS) using an ExionLC™ UHPLC system coupled online to a SCIEX QTRAP 6500+ triple quadrupole mass spectrometer (SCIEX, Canada). LC-MS parameters, such as the LC gradient and MRM parameters (Q1 and MRM scans) were adapted and optimized based on the previous studies [11,12].

The loaded sample volume was 10 μL per injection. HPLC separation was carried out using Zorbax Eclipse Plus RRHD C18 RP-UHPLC (150 X 2.1 mm i.d., 1.8 μm particles; Agilent Technologies) with gradient elution. Mobile phase A was 0.1% FA in water; mobile phase B was 0.1% FA in acetonitrile. LC separation was performed at a flow rate of 0.4 mL/min using a 53 min gradient from 2 to 45% of mobile phase B. Mass-spectrometric measurements were carried out using the MRM acquisition method. The electrospray ionization (ESI) source settings were as follows: ion spray voltage 4000 V, temperature 450°C, ion source gas 40 L/min. The corresponding transition list for MRM experiments with Q1;Q3 masses for each peptide is available in Table S1.

For quantitative analysis of LC-MS/MS raw data Skyline Quantitative Analysis software (version 20.2.0.343, University of Washington, USA) was used [46,47]. To calculate the protein levels in the measured samples calibration curves were generated using 1/(X×X)-weighted linear regression methods.

The MRM data quality was checked manually in Skyline for all selected proteins/peptides and includes the absence of interference peaks and the good quality of the peak shape, and the ratios of the precursor and product ion. The exemplary MRM data (from Skyline) for selected proteins are presented in Figures S1-S3.

All experimental results from MRM analysis were uploaded to the PeptideAtlas SRM Experiment Library (PASSEL) and are available via link:

http://www.peptideatlas.org/PASS/PASS04817 (accessed on 15 March 2023)

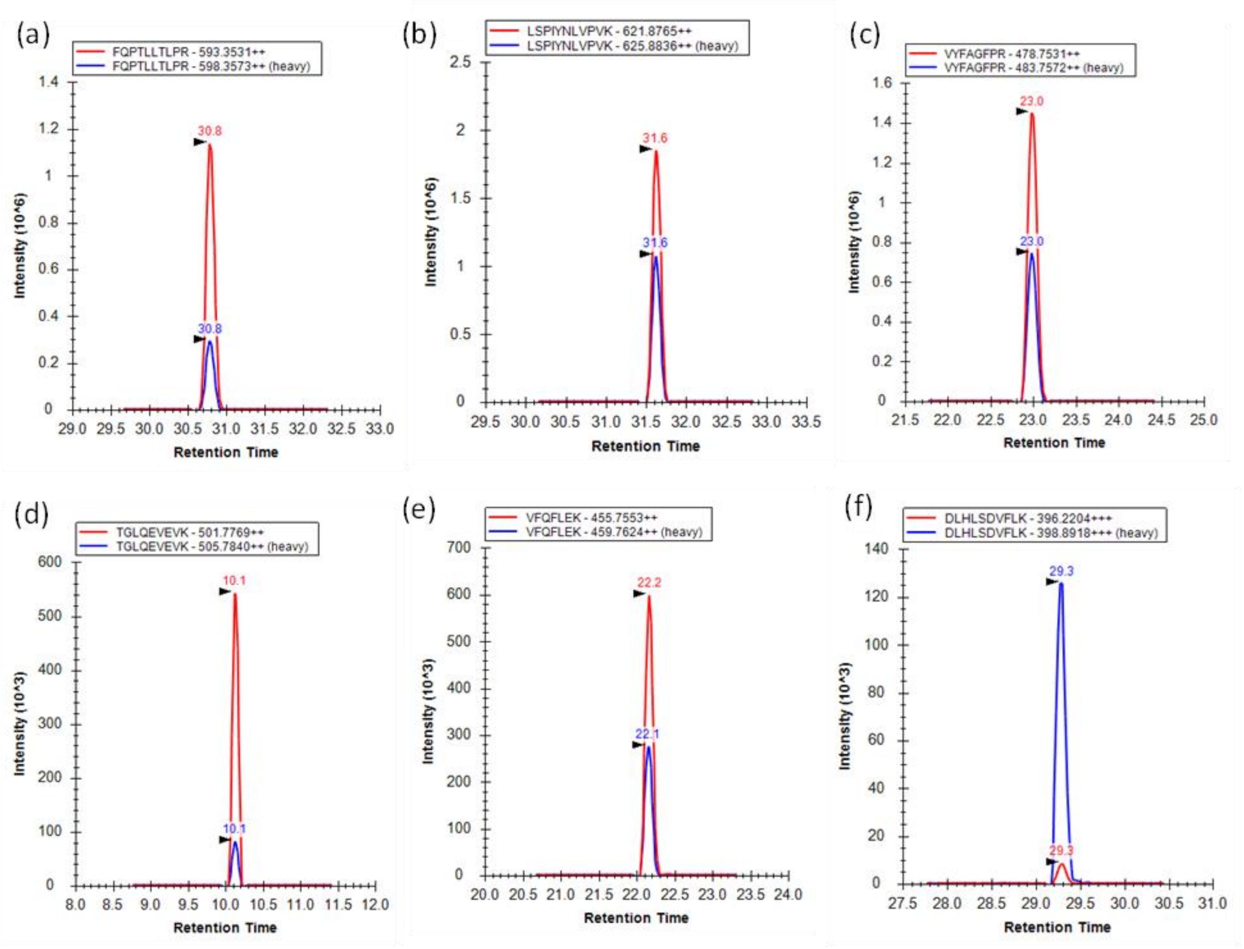

Figure S1. The exemplary MRM data (from Skyline) for selected proteins are presented: (**a**) Plasma protease C1 inhibitor; (**b**) Complement component C9; (**c**) Vitamin K-dependent protein S; (**d**) Complement C3; (**e**) Complement C5; (**f**) Complement component C6.

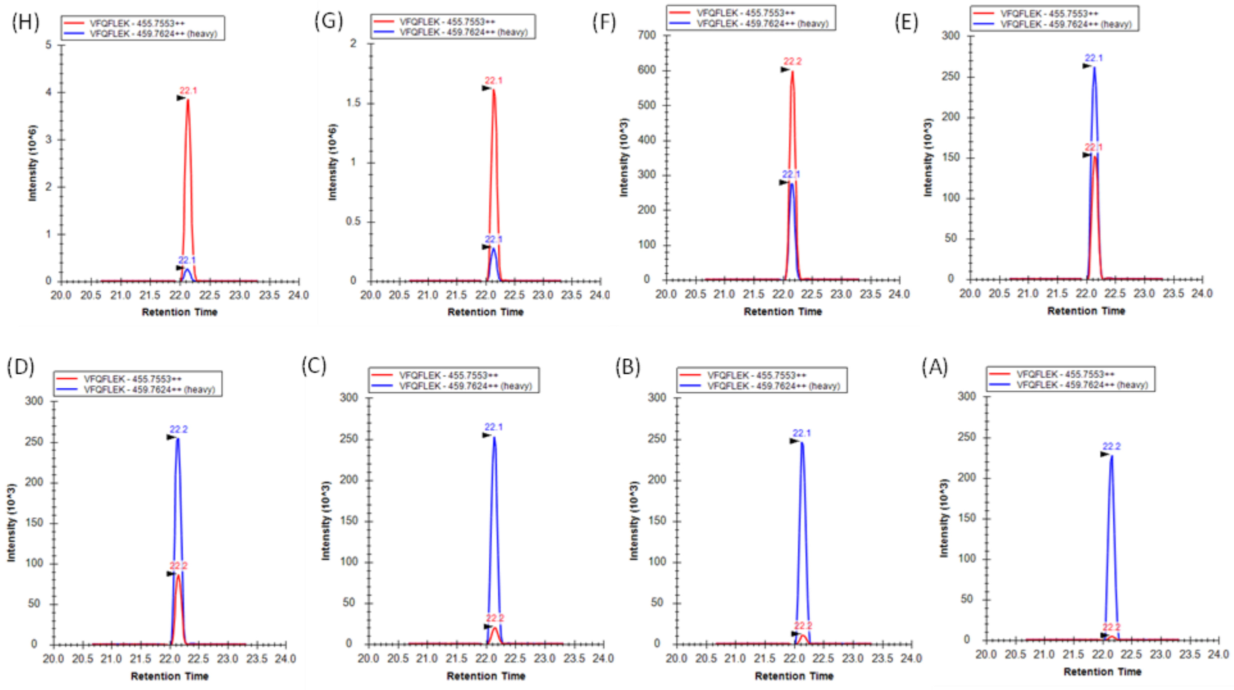

Figure S2. The exemplary MRM data (from Skyline) for calibration curve for Complement C5. (**A-H**) - points on the standard curve.

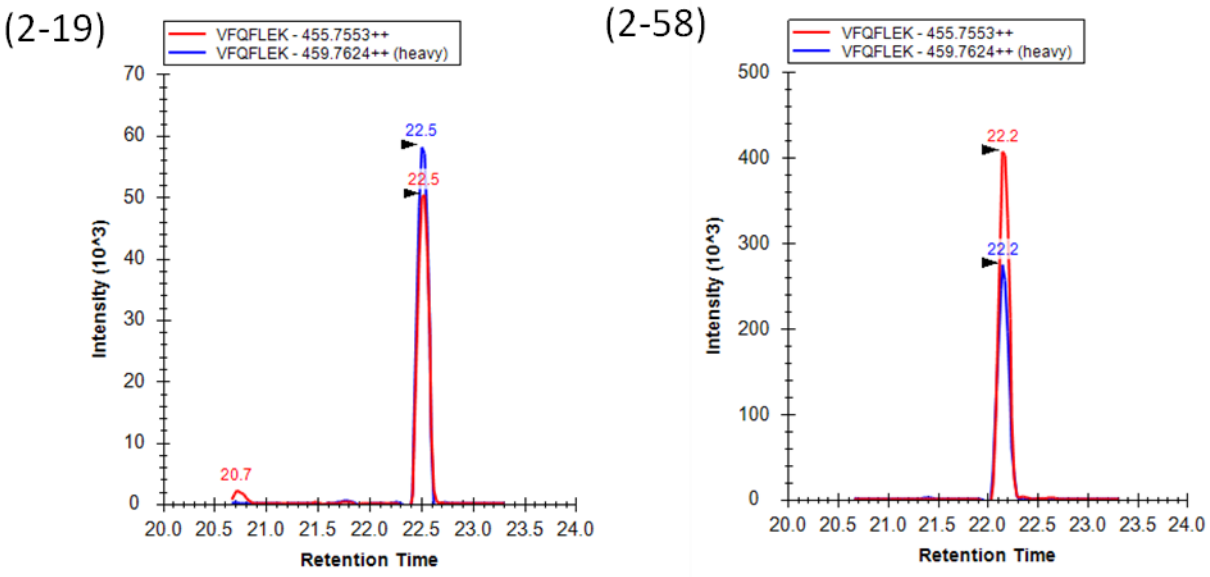

Figure S3. The exemplary MRM data (from Skyline) for Complement C5 analysis in blood plasma samples from two selected patients. 2-19, 2-58 - patient codes.

Table S1. Transition list for MRM experiments with Q1;Q3 masses for each NAT and SIS peptide.

Protein name	Uniprot ID	Gene name	Peptide sequence	Light		Heavy	
				Precursor m/z	Product m/z	Precursor m/z	Product m/z
60 kDa heat shock protein mitochondrial	P10809	HSPD1	GIIDPTK	372.22	573.32	376.23	581.34
72 KDa type IV collagenase	P08253	MMP2	IDAVYEAPQEEK	696.34	993.45	700.35	1001.47
78 kDa glucose-regulated protein	P11021	HSPA5	ITPSYVAFTPEGER	783.89	676.83	788.90	681.83
A disintegrin and metalloproteinase with thrombospondin motifs 2	O95450	ADAMTS2	IILLSYGK	453.79	680.40	457.79	688.41
A disintegrin and metalloproteinase with thrombospondin motifs 20	P59510	ADAMTS20	IPAGATNVDIR	563.81	288.20	568.82	298.21
A disintegrin and metalloproteinase with thrombospondin motifs 9	Q9P2N4	ADAMTS9	LYNPDVR	438.73	600.31	443.74	610.32
Actin, aortic smooth muscle	P62736|P68032|8others	ACTA2 | ACTC1 | 8 others	SYELPDGQVITIGNER	895.95	380.15	900.95	380.15
Adhesion G protein-coupled receptor F5	Q8IZF2	ADGRF5	DVIVHPLPLK	377.57	458.80	380.24	462.81
Adipocyte plasma membrane-associated protein	Q9HDC9	APMAP	LLEYDTVTR	555.30	883.42	560.30	893.42
Adiponectin	Q15848	ADIPOQ	IFYNQQNHYDGSTGK	591.27	756.33	593.94	760.34
ADM	P35318	ADM	LDVASEFR	468.74	538.26	473.75	548.27
Afamin	P43652	AFM	DADPDTFFAK	563.76	825.41	567.76	833.43
Alpha-1-acid glycoprotein 1	P02763	ORM1	NWGLSVYADKPETTK	570.29	704.87	572.96	708.88
Alpha-1-antichymotrypsin	P01011	SERPINA3	EIGELYLPK	531.30	244.17	535.30	252.18
Alpha-1-antitrypsin	P01009	SERPINA1	SVLGQLGITK	508.31	415.26	512.32	419.27
Alpha-1B-glycoprotein_VAR_018369	P04217	A1BG	LETPDFQLFK	619.33	243.13	623.33	243.13
Alpha-2-antiplasmin	P08697	SERPINF2	LGNQEPGGQTALK	656.85	771.44	660.85	779.45
Alpha-2-HS-glycoprotein	P02765	AHSG	FSVVYAK	407.23	579.35	411.24	587.36
Alpha-2-macroglobulin	P01023	A2M	AIGYLNTGYQR	628.33	851.44	633.33	861.45
Angiogenin	P03950	ANG	DINTFIHGNK	386.87	465.74	389.54	469.75
Angiopoietin-related protein 3	Q9Y5C1	ANGPTL3	DLVFSTWDHK	416.54	510.25	419.21	514.26
Angiotensinogen	P01019	AGT	ALQDQLVLVAAK	634.88	542.82	638.89	546.83
Antithrombin-III	P01008	SERPINC1	DDLYVSDAFHK	437.21	483.74	439.88	487.74
Apolipoprotein A-I	P02647	APOA1	ATEHLSTLSEK	405.88	572.80	408.55	576.80
Apolipoprotein A-II	P02652	APOA2	SPELQAEAK	486.75	443.24	490.76	447.24
Apolipoprotein A-IV	P06727	APOA4	LGEVNTYAGDLQK	704.36	300.16	708.37	300.16
Apolipoprotein B-100	P04114	APOB	FPEVDVLTK	524.29	450.76	528.30	454.76
Apolipoprotein C-I	P02654	APOC1	EWFSETFQK	601.28	886.43	605.29	894.44

Apolipoprotein C-II	P02655	APOC2	TYLPAVDEK	518.27	265.12	522.28	265.12
Apolipoprotein C-III	P02656	APOC3	GWVTDGFSSLK	598.80	244.11	602.81	244.11
Apolipoprotein C-IV	P55056	APOC4	ELLETVVNR	536.80	717.39	541.81	727.40
Apolipoprotein D	P05090	APOD	NILTSNNIDVK	615.84	228.13	619.85	228.13
Apolipoprotein E	P02649	APOE	LGPLVEQGR	484.78	399.73	489.78	404.73
Apolipoprotein F	Q13790	APOF	SGVQQLIQYYQ DQK	566.62	613.33	569.29	613.33
Apolipoprotein L1	O14791	APOL1	VAQELEEK	473.25	775.38	477.26	783.40
Apolipoprotein M	O95445	APOM	AFLLTPR	409.25	599.39	414.25	609.40
Apolipoprotein(a)	P08519	LPA	GTYSTTVTGR	521.76	721.38	526.77	731.39
Aromatase	P11511	CYP19A1	NMLEMIFTPR	626.31	893.45	631.32	903.46
Atrial natriuretic peptide receptor 1	P16066	NPR1	ITDYGLESFR	600.80	986.46	605.80	996.47
Attractin	O75882	ATRN	SVNNVVVR	443.76	700.41	448.76	710.42
Autism susceptibility gene 2 protein	Q8WXX7	AUTS2	ALSLASSSGSDK	561.79	738.33	565.79	746.34
B-cell scaffold protein with ankyrin repeats	Q8NDB2	BANK1	LTIVHHPGGK	353.54	316.67	356.21	320.67
Beta-2-glycoprotein 1	P02749	APOH	ATVVYQGER	511.77	751.37	516.77	761.38
Beta-2-microglobulin	P61769	B2M	VNHVTLSQPK	374.88	459.26	377.55	467.27
Beta-Ala-His dipeptidase	Q96KN2	CNDP1	ALEQDLPVNIK	620.35	570.36	624.36	578.38
Beta-nerve growth factor	P01138	NGF	TTATDIK	375.21	547.31	379.21	555.32
Biotinidase	P43251	BTD	SHLIIAQVAK	360.56	451.27	363.23	451.27
C4b-binding protein alpha chain	P04003	C4BPA	EDVYVVGTVLR	625.34	545.34	630.35	555.35
Cadherin-13	P55290	CDH13	INENTGSVSVTR	638.83	228.13	643.83	228.13
Cadherin-5	P33151	CDH5	ELDSTGTPTGK	553.27	863.41	557.28	871.42
Calcitonin gene-related peptide 1	P06881	CALCA	NNFVPTNVGSK	588.80	702.38	592.81	710.39
Calcitonin	P01258	CALCA	FHTFPQTAIGVG APGK	543.29	585.34	545.96	593.35
Calponin-1	P51911	CNN1	VNVGVK	308.19	516.31	312.20	524.33
Carbonic anhydrase 1	P00915	CA1	VLDALQAIK	485.80	758.44	489.81	766.45
Carboxypeptidase B2	Q96IY4	CPB2	IAWHVIR	298.85	262.67	302.18	267.67
Carboxypeptidase N catalytic chain	P15169	CPN1	SIPQVSPVR	491.79	391.73	496.79	396.73
Carboxypeptidase N subunit 2	P22792	CPN2	AGGSWDLAVQE R	644.82	830.44	649.82	840.44
Cartilage acidic protein 1	Q9NQ79	CRTAC1	GVASLFAGR	439.25	721.40	444.25	731.41
Cathelicidin antimicrobial peptide	P49913	CAMP	AIDGINQR	443.74	702.35	448.74	712.36
Cation-independent mannose-6-phosphate receptor	P11717	IGF2R	GHQAFDVGQPR	404.54	457.25	407.87	467.26
CD40 ligand	P29965	CD40LG	SQFEGFVK	471.24	726.38	475.25	734.40
CD44 antigen	P16070	CD44	YGFIEGHVVIPR	462.92	612.35	466.26	617.35
CD5 antigen-like	O43866	CD5L	LVGGLHR	376.23	213.16	381.24	213.16
Gelsolin	P06396	GSN	EGGQTAPASTR	537.76	531.29	542.77	541.30
Ceruloplasmin	P00450	CP	IYHSHIDAPK	394.21	452.74	396.88	456.74

Cholesteryl ester transfer protein	P11597	CETP	GVSLFDIINPEIIT R	843.97	842.47	848.98	852.48
Cholinesterase	P06276	BCHE	YLTLNTESTR	599.31	921.46	604.31	931.47
Chromogranin-A	P10645	CHGA	ELQDLALQGAK	593.33	815.46	597.33	823.48
Claudin-5	O00501	CLDN5	PDLSFPVK	451.75	805.45	455.76	813.46
Clusterin	P10909	CLU	ELDESLQVAER	644.82	375.20	649.83	385.21
Coagulation factor IX	P00740	F9	SALVLQYLR	531.82	692.41	536.82	702.42
Coagulation factor V	P12259	F5	AEVDDVIQVR	572.30	844.45	577.31	854.46
Coagulation factor VII	P08709	F7	VSQYIEWLQK	647.35	979.52	651.35	987.54
Coagulation factor VIII	P00451	F8	LHPTHYSIR	375.21	437.23	378.54	442.24
Coagulation factor X	P00742	F10	MLEVPYVDR	561.29	649.33	566.29	659.34
Coagulation factor XI	P03951	F11	TSESGLPSTR	517.76	460.25	522.76	470.26
Coagulation factor XII	P00748	F12	EQPPSLTR	464.25	335.70	469.25	340.70
Coagulation factor XIII A chain	P00488	F13A1	GTYIPVPIVSELQ SGK	844.47	435.22	848.47	435.22
Coagulation factor XIII B chain	P05160	F13B	IQTHSTTYR	369.52	627.31	372.86	637.32
Collagen alpha-1(I) chain	P02452	COL1A1	GVVGLPGQR	441.76	457.25	446.77	467.26
Collagen alpha-1(III) chain	P02461	COL3A1	GGAGPPGPEGG K	490.74	369.69	494.75	373.70
Collagen alpha-1(XVIII) chain	P39060	COL18A1	AVGLAGTFR	446.26	721.40	451.26	731.41
Collagen alpha-2(I) chain	P08123	COL1A2	GVVGPQGAR	420.74	342.69	425.74	347.70
Complement C1q subcomponent subunit A	P02745	C1QA	PAFSAIR	381.22	593.34	386.22	603.35
Complement C1q subcomponent subunit B	P02746	C1QB	IAFSATR	383.22	581.30	388.22	591.31
Complement C1q subcomponent subunit C	P02747	C1QC	FQSVFTVTR	542.79	809.45	547.80	819.46
Complement C1r subcomponent	P00736	C1R	GLTLHLK	261.17	306.20	263.84	310.20
Complement C1r subcomponent-like protein	Q9NZP8	C1RL	VVVHPDYR	328.85	443.23	332.18	448.24
Complement C1s subcomponent	P09871	C1S	TNFDNDIALVR	639.33	216.10	644.33	216.10
Complement C2	P06681	C2	HAFILQDTK	536.79	864.48	540.80	872.50
Complement C3	P01024	C3	TGLQEVEVK	501.78	731.39	505.78	739.41
Complement C4	P0C0L4|P0C0L5	C4A | C4B	VGDTLNLNLR	557.81	629.37	562.82	639.38
Complement C5	P01031	C5	VFQFLEK	455.76	664.37	459.76	672.38
Complement C6	P13671	C6	DLHLSDVFLK	396.22	411.24	398.89	415.25
Complement C7	P10643	C7	AASGTQNNVLR	565.80	494.76	570.80	499.77
Complement C8 alpha chain	P07357	C8A	MESLGITSR	497.26	733.42	502.26	743.43
Complement C8 beta chain	P07358	C8B	SDLEVAHYK	354.51	430.24	357.18	434.24
Complement C9	P02748	C9	LSPIYNLVPVK	621.88	521.82	625.88	525.83
Complement factor B	P00751	CFB	EELLPAQDIK	578.32	671.37	582.32	679.39
Complement factor D	P00746	CFD	THHDGAITER	379.52	405.21	382.86	415.22

Complement factor H	P08603	CFH	SSQESYAHGTK	398.85	510.74	401.52	514.75
Complement factor I	P05156	CFI	VFSLQWGEVK	596.82	946.50	600.83	954.51
Corticosteroid-binding globulin	P08185	SERPINA6	WSAGLTSSQVD LYIPK	882.96	244.17	886.97	252.18
C-reactive protein	P02741	CRP	AFVFPK	354.71	244.17	358.71	252.18
Creatine kinase B-type	P12277	CKB	DLFDPIIEDR	616.81	742.41	621.82	752.42
Creatine kinase M-type	P06732	CKM	FEEILTR	454.25	631.38	459.25	641.39
Cystatin-C	P01034	CST3	ALDFAVGEYNK	613.81	300.16	617.81	300.16
Desmoplakin	P15924	DSP	AELIVQPELK	570.34	486.29	574.34	494.31
Dickkopf-related protein 1 and 2	O94907|Q9UBU2	DKK1 | DKK2	GSHGLEIFQR	381.87	450.25	385.20	460.25
Di-N-acetylchitobiase	Q01459	CTBS	ATYIQNYR	514.76	428.72	519.77	433.72
Elastin	P15502	ELN	LPGGYGLPYTTG K	662.35	605.81	666.36	609.82
Endothelial lipase	Q9Y5X9	LIPG	LVSALHTR	299.52	342.69	302.85	347.70
Endothelial protein C receptor	Q9UNN8	PROCR	TLAFPLTIR	516.32	817.49	521.32	827.50
Epidermal growth factor receptor	P00533	EGFR	IPLENLQIIR	604.87	548.33	609.88	553.33
E-selectin	P16581	SELE	YTHLVAIQNK	396.22	402.18	398.90	402.18
Extracellular matrix protein 1	Q16610	ECM1	NVALVSGDTEN AK	659.34	821.36	663.34	829.38
Fatty acid-binding protein heart	P05413	FABP3	SLGVGFATR	454.25	707.38	459.26	717.39
Ferritin heavy chain	P02794	FTH1	NVNQSLLELHK	432.24	541.30	434.91	545.31
Ferritin light chain	P02792	FTL	LGGPEAGLGEY LFER	804.41	913.44	809.41	923.45
Fetuin-B	Q9UGM5	FETUB	LVVLPFPK	456.80	700.44	460.81	708.45
Fibrinogen alpha chain	P02671	FGA	VQHIQLLQK	553.84	228.13	557.84	228.13
Fibrinogen beta chain	P02675	FGB	HQLYIDETVNSN IPTNLR	709.70	764.38	713.04	764.38
Fibrinogen gamma chain	P02679	FGG	YEASILTHDSSIR	497.92	600.32	501.26	605.33
Fibronectin	P02751	FN1	HTSVQTTSSGSG PFTDVR	621.97	734.38	625.30	744.39
Fibulin-1	P23142	FBLN1	TGYYFDGISR	589.78	857.42	594.78	867.42
Ficolin-2	Q15485	FCN2	GTHGSFANGIN WK	463.56	447.24	466.23	455.25
Ficolin-3	O75636	FCN3	YAVSEAAAHK	349.51	357.18	352.19	361.19
Follistatin-related protein 1 | Early endosome antigen 1	Q12841|Q15075	FSTL1 | EEA2	YVQELQK	454.25	645.36	458.25	653.37
Fructose-bisphosphate aldolase B	P05062	ALDOB	ALQASALAAWG GK	622.34	530.28	626.35	534.29
Galectin-3	P17931	LGALS3	IALDFQR	431.74	678.36	436.75	688.37
Galectin-3-binding protein	Q08380	LGALS3BP	SDLAVPSELALL K	678.39	870.53	682.40	878.54
Gamma-enolase	P09104	ENO2	YITGDQLGALY QDFVR	620.32	827.40	623.65	837.41

Gelsolin	P06396	GSN	AGALNSNDAFVLK	660.35	200.10	664.36	200.10
Glial fibrillary acidic protein	P14136	GFAP	LADVYQAELR	589.31	300.16	594.32	300.16
Glutamate receptor ionotropic NMDA 2A	Q12879	GRIN2A	FSYIPEAK	477.75	444.25	481.76	452.26
Glutamate receptor ionotropic NMDA 2B	Q13224	GRIN2B	EPGGPSFTIGK	545.28	204.13	549.29	212.15
Glutathione peroxidase 3	P22352	GPX3	QEPGENSEILPTLK	777.90	649.35	781.91	653.36
Glutathione S-transferase P	P09211	GSTP1	TLGLYGK	376.22	537.30	380.23	545.32
Haptoglobin	P00738	HP	DIAPTLTLYVGK	645.87	496.29	649.88	500.30
Heat shock protein beta-1	P04792	HSPB1	LFDQAFGLPR	582.31	903.47	587.32	913.48
Hemoglobin subunit alpha	P69905	HBA1;	VGAHAGEYGAEALER	510.58	488.28	513.92	498.29
Hemopexin	P02790	HPX	NFPSPVDAAFR	610.81	480.25	615.81	485.26
Heparin cofactor 2	P05546	SERPIND1	TLEAQLTPR	514.79	814.44	519.79	824.45
Hepatocyte growth factor-like protein	P26927	MST1	SPLNDFQVLR	594.82	891.47	599.83	901.48
Histidine-rich glycoprotein	P04196	HRG	ADLFYDVEALDLESPK	912.95	331.20	916.96	339.21
Hornerin	Q86YZ3	HRNR	GSGSGQSPSSGQHGTGFGR	583.26	730.34	586.60	735.34
Hyaluronan-binding protein 2	Q14520	HABP2	VVLGDQDLK	493.78	788.41	497.79	796.43
Ig gamma-1 chain C region	P01857	IGHG1	GPSVFPLAPSSK	593.83	846.47	597.83	854.49
Ig mu chain C region	P01871	IGHM	GFPSVLR	388.23	286.18	393.23	291.19
Ig mu heavy chain disease protein and Ig mu chain C	P04220|P01871	IGHM	VSVFVPPR	450.77	615.36	455.77	625.37
IgGFc-binding protein	Q9Y6R7	FCGBP	GATTSPGVYELSSR	712.85	1007.52	717.86	1017.52
Immunoglobulin kappa variable 4-1	P06312	IGKV4-1	NYLAWYQQKPGQPPK	606.65	770.92	609.32	774.92
Insulin-like growth factor I	P05019	IGF1	GFYFNKPTGYGSSSR	556.60	732.35	559.93	737.35
Insulin-like growth factor-binding protein 1	P08833	IGFBP1	ALPGEQQPLHALTR	510.95	597.35	514.29	607.35
Insulin-like growth factor-binding protein 2	P18065	IGFBP2	LIQGAPTIR	484.80	614.36	489.80	624.37
Insulin-like growth factor-binding protein 3	P17936	IGFBP3	FLNVLSPR	473.28	685.40	478.28	695.41
Insulin-like growth factor-binding protein complex acid labile subunit	P35858	IGFALS	LAELPADALGPLQR	732.41	1037.57	737.42	1047.58
Inter-alpha-trypsin inhibitor heavy chain H1	P19827	ITIH1	GSLVQASEANLQAAQDFVR	668.68	806.42	672.01	816.42
Inter-alpha-trypsin inhibitor heavy chain H2	P19823	ITIH2	SLAPTAAAK	415.24	558.32	419.25	566.34
Inter-alpha-trypsin inhibitor heavy chain H4	Q14624	ITIH4	SPEQQETVLDGNLIIR	604.65	685.44	607.99	695.44

Intercellular adhesion molecule 1	P05362	ICAM1	LLGIETPLPK	540.84	854.50	544.84	862.51
Interleukin-10	P22301	IL10	AHVNSLGENLK	394.55	560.30	397.22	568.32
Interleukin-6	P05231	IL6	FESSEEQAR	541.74	806.36	546.75	816.37
Interstitial collagenase	P03956	MMP1	AFQLWSNVTPL TFTK	876.97	347.17	880.98	347.17
Kallistatin	P29622	SERPINA4	VGSALFLSHNLK	429.25	593.83	431.92	597.84
Keratin type I cytoskeletal 10	P13645	KRT10	SLLEGEGSSGGG GR	631.80	201.12	636.81	201.12
Keratin type I cytoskeletal 9	P35527	KRT9	TLLDIDNTR	530.79	846.43	535.79	856.44
Keratin-type II cytoskeletal 2 epidermal	P35908	KRT2	YEELQVTVGR	597.31	293.11	602.32	293.11
Kininogen-1	P01042	KNG1	DIPTNSPELEETL THTITK	713.70	637.66	716.37	640.33
Lactotransferrin	P02788	LTF	YLGPQYVAGITN LK	768.92	602.34	772.93	606.35
Leucine-rich alpha-2-glycoprotein	P02750	LRG1	DLLLPQPDLR	590.34	725.39	595.34	735.40
Lipopolysaccharide-binding protein	P18428	LBP	ITLPDFTGDLR	624.34	920.45	629.34	930.46
L-selectin	P14151	SELL	AEIEYLEK	497.76	552.30	501.77	560.32
Lumican	P51884	LUM	SLEDLQLTHNK	433.23	549.28	435.90	553.29
Lysozyme C	P61626	LYZ	AWVAWR	394.71	531.30	399.72	541.31
Mannan-binding lectin serine protease 1	P48740	MASP1	TGVITSPDFPNP YPK	816.92	258.14	820.92	258.14
Mannan-binding lectin serine protease 2A	O00187	MASP2	WPEPVFGR	494.26	575.33	499.26	585.34
Mannose-binding protein C	P11226	MBL2	WLTFSLGK	476.27	652.37	480.28	660.38
Matrix Gla protein	P08493	MGP	NANTFISPQQR	638.33	728.40	643.33	738.41
Matrix metalloproteinase-9	P14780	MMP9	AVIDDAFAR	489.26	807.40	494.26	817.41
Melanotransferrin	P08582	MELTF	YYDYSGAFR	571.25	815.37	576.26	825.38
Metalloproteinase inhibitor 1	P01033	TIMP1	GFQALGDAADI R	617.31	517.28	622.32	517.28
Metalloproteinase inhibitor 2	P16035	TIMP2	EYLIAGK	397.23	275.17	401.23	283.19
Metalloproteinase inhibitor 4	Q99727	TIMP4	VVPASADPADT EK	650.32	551.26	654.33	555.26
Microtubule-associated protein tau	P10636	MAPT	EADLPEPSEK	557.77	686.34	561.77	694.35
Mucin-16	Q8WXI7	MUC16	ELGPYTLDR	532.27	504.28	537.28	514.29
Myelin basic protein	P02686 (not in isoform 2)		GVDAQGTLSK	488.26	819.42	492.27	827.43
Myeloblastin	P24158	PRTN3	LVNVVLGAHNV R	430.93	539.81	434.26	544.81
Myeloperoxidase	P05164	MPO	VFFASWR	456.74	666.34	461.74	676.34
N(G),N(G)-dimethylarginine dimethylaminohydrolase 1	O94760	DDAH1	TPEEYPESAK	575.77	525.24	579.77	529.25
N-acetylmuramoyl-L-alanine amidase	Q96PD5	PGLYRP2	AGLLRPDYALL GHR	517.96	482.28	521.30	492.29
Natriuretic peptides B	P16860	NPPB	EVATEGIR	437.74	646.35	442.74	656.36

Neuropilin-2	O60462	NRP2	ALQVVR	343.22	501.31	348.23	511.32
Neutrophil gelatinase-associated lipocalin	P80188	LCN2	ITLYGR	361.71	508.29	366.72	518.30
Nucleoside diphosphate kinase A, B	P15531	NME1	PFFAGLVK	439.76	634.39	443.77	642.41
Occludin	Q16625	OCLN	SLQSELDEINK	638.32	947.47	642.33	955.48
Osteopontin	P10451	SPP1	GDSVVYGLR	483.26	607.36	488.26	617.36
Oxidized low-density lipoprotein receptor 1	P78380	OLR1	LEGQISAR	437.24	631.35	442.25	641.36
Pappalysin-1	Q13219	PAPPA	AYLDVNELK	532.78	235.11	536.79	235.11
Peroxiredoxin-1	Q06830	PRDX1	ADEGISFR	447.72	409.22	452.72	419.23
Peroxiredoxin-2	P32119	PRDX2	GLFIIDGK	431.76	692.40	435.76	700.41
Phosphatidylcholine-sterol acyltransferase	P04180	LCAT	SSGLVSNAPGVQ IR	692.88	669.40	697.88	679.41
Phosphatidylinositol-glycan-specific phospholipase D	P80108	GPLD1	FGSSLITVR	490.28	488.32	495.29	498.33
Phospholipid transfer protein	P55058	PLTP	AVEPQLQEEER	664.33	514.75	669.33	519.76
Pigment epithelium-derived factor	P36955	SERPINF1	LQSLFDSPDFSK	692.34	1142.54	696.35	1150.55
Plasma protease C1 inhibitor	P05155	SERPING1	FQPTLLTLPR	593.35	910.57	598.36	920.58
Plasma serine protease inhibitor	P05154	SERPINA5	GFQQLLQELNQP R	524.28	514.27	527.62	524.28
Plasminogen activator inhibitor 1	P05121	SERPINE1	VFQQVAQASK	553.30	504.28	557.31	512.29
Plasminogen	P00747	PLG	EAQLPVIENK	570.82	699.40	574.82	707.42
Plastin-2	P13796	LCP1	ISFDEFIK	499.76	798.40	503.77	806.42
Platelet endothelial cell adhesion molecule	P16284	PECAM1	SELVTVTESFSTP K	762.89	217.08	766.90	217.08
Platelet glycoprotein VI	Q9HCN6	GP6	EGDPAPYK	438.71	407.23	442.72	415.24
Platelet-activating factor acetylhydrolase	Q13093	PLA2G7	GSVHQNFADFTF ATGK	576.28	886.43	578.95	894.44
Pregnancy zone protein	P20742	PZP	ISEITNIVSK	552.32	774.47	556.33	782.49
Proenkephalin-A	P01210	PENK	ELLETGDNR	523.76	562.26	528.76	572.27
Prolactin	P01236	PRL	IDNYLK	383.21	652.33	387.22	660.34
Protein AMBP	P02760	AMBP	HHGPTITAK	321.18	275.13	323.85	275.13
Protein S100-A12	P80511	S100A12	GHFDTLSK	302.16	234.14	304.83	242.16
Protein S100-A9	P06702	S100A9	DLQNFLK	439.24	649.37	443.25	657.38
Protein S100-B	P04271	S100B	EQEVVDK	423.71	460.28	427.72	468.29
Protein Z-dependent protease inhibitor	Q9UK55	SERPINA10	ETSNFGFSLLR	635.82	692.41	640.83	702.42
Protein_deglycase_DJ-1	Q99497	PARK7	ALVILAK	364.26	543.39	368.26	551.40
Proteoglycan 4	Q92954	PRG4	DQYYNIDVPSR	685.32	244.09	690.33	244.09
Prothrombin	P00734	F2	ELLESYIDGR	597.80	710.35	602.81	720.36
P-selectin	P16109	SELP	TWTWVGTK	489.76	691.38	493.76	699.39
Ras GTPase-activating protein nGAP	Q9UJF2	RASAL2	ETQSTPQSAPQV R	714.86	882.48	719.86	892.49
Resistin	Q9HD89	RETN	IQEVAGSLIFR	616.85	242.15	621.86	242.15

Retinol-binding protein 4	P02753	RBP4	YWGVASFLQK	599.82	849.48	603.82	857.50
Serotransferrin	P02787	TF	DGAGDVAFVK	489.75	735.40	493.76	743.42
Serum albumin	P02768 (not in isoform 2)		LVNEVTEFAK	575.31	218.15	579.32	226.16
Serum amyloid A-1 and A-2 proteins	P0DJI8|P0DJI9	SAA2	EANYIGSDK	498.74	406.19	502.74	414.21
Serum amyloid A-4 protein	P35542	SAA4	GNYDAAQR	447.71	445.25	452.71	455.26
Serum amyloid P-component	P02743	APCS	IVLGQEQDSYGGK	697.35	591.28	701.36	595.28
Serum paraoxonase/arylesterase 1	P27169	PON1	IFFYDSENPPASEVLR	942.46	868.49	947.47	878.50
Serum paraoxonase/lactonase 3	Q15166	PON3	ILIGTVFHK	343.21	401.23	345.89	405.24
Sex hormone-binding globulin	P04278	SHBG	TSSSFEVR	456.72	724.36	461.73	734.37
SPARC	P09486	SPARC	LEAGDHPVELLAR	473.92	589.32	477.26	594.32
Spermine oxidase	Q9NWM0	SMOX	YYSTTHGALLSGQR	518.60	614.33	521.93	619.33
Sterile alpha motif domain-containing protein 9-like	Q8IVG5	SAMD9L	ENVLDEVANAK	601.31	859.45	605.31	867.47
Stromelysin-1	P08254	MMP3	TYFFVEDK	524.75	784.39	528.76	792.40
Target of Nesh-SH3	Q7Z7G0	ABI3BP	IYLSDSLTGK	548.80	277.15	552.80	277.15
TBC1 domain family member 10A	Q9BXI6	TBC1D10A	YLPGYYSEK	560.27	422.20	564.28	426.20
Tenascin	P24821	TNC	FTTDLDSPR	526.26	803.39	531.26	813.40
Tenascin-X	P22105|Q16473	TNXB | TNXA	ILISGLEPSTPYR	723.40	720.37	728.41	730.38
Tetranectin	P05452	CLEC3B	NWETEITAQPDGGK	773.36	473.24	777.37	481.25
Thrombomodulin	P07204	THBD	SSVAADVISLLLNGDGGVGR	634.01	731.34	637.35	741.35
Thrombospondin-1	P07996	THBS1	GTLLALER	436.76	488.28	441.77	498.29
Thrombospondin-4	P35443	THBS4	KPQDFLEELK	416.23	518.28	418.90	526.30
Thyroglobulin	P01266	TG	FSPDDSAGASALLR	703.85	586.80	708.85	591.80
Thyroxine-binding globulin	P05543	SERPINA7	AVLHIGEK	289.51	348.71	292.18	352.71
Tissue_factor_pathway_inhibitor	P10646	TFPI	FYYNSVIGK	545.78	780.43	549.79	788.44
Tissue-type plasminogen activator	P00750	PLAT	VVPGEEEQK	507.76	816.37	511.77	824.39
Transcription factor SOX-1	P35716	SOX11	AAQSGDYGGAGDDYVLGSLR	657.97	545.34	661.31	555.35
Transferrin receptor protein 1	P02786	TFRC	GFVEPDHYVVVGAQR	558.29	734.88	561.62	739.88
Transthyretin	P02766	TTR	GSPAINVAVHVFR	456.26	611.86	459.59	616.86
Tumor necrosis factor receptor superfamily member 1A	P19438	TNFRSF1A	LGLSDHEIDR	385.53	521.25	388.87	526.26
Tumor necrosis factor receptor superfamily member 1B	P20333	TNFRSF1B	DEQVPFSK	475.23	478.27	479.24	486.28

Vascular cell adhesion protein 1	P19320	VCAM1	NTVISVNPSTK	580.32	845.47	584.33	853.49
Vascular endothelial growth factor B	O43915|P49765|P15692	VEGFD | VEGFB | VEGFA	VVSWIDVYTR	619.33	1039.52	624.34	1049.53
Vascular endothelial growth factor D	O43915	VEGFD	DLIQHPK	425.74	622.37	429.75	630.38
Vascular non-inflammatory molecule 3	Q9NY84	VNN3	TETPVSK	381.21	231.10	385.21	231.10
Vasorin	Q6EMK4	VASN	YLQGSSVQLR	575.81	746.42	580.82	756.42
Vitamin D-binding protein	P02774	GC	VLEPTLK	400.25	587.34	404.26	595.35
Vitamin K-dependent protein C	P04070	PROC	LGEYDLR	433.22	752.36	438.23	762.37
Vitamin K-dependent protein S	P07225	PROS1	VYFAGFPR	478.75	694.37	483.76	704.38
Vitamin K-dependent protein Z	P22891	PROZ	DFAEHLLIPR	404.22	474.78	407.56	479.79
Vitronectin	P04004	VTN	FEDGVLDPDYPR	711.83	647.31	716.83	657.32
von Willebrand factor	P04275	VWF	ILAGPAGDSNVVK	620.85	472.25	624.86	476.25
Xaa-Pro dipeptidase	P12955	PEPD	AVYEAVLR	460.76	750.41	465.77	760.42
Zinc-alpha-2-glycoprotein	P25311	AZGP1	EIPAWVPFDPAAQITK	891.97	1087.58	895.98	1095.59

Table S2. Comparison of studied hemostasis parameters between study groups.

Parameter	AMI control	AMI post-COVID	Control	Control post-COVID	*p* adj. AMI control vs AMI post-COVID	*p* adj. AMI control vs Control	*p* adj. AMI post-COVID vs Control post-COVID	*p* adj. AMI post-COVID vs Control
A10, mm	48 [41; 55]	42 [36; 51]	42 [38; 48]	42 [37; 47.5]	0.2051	0.1008	0.945	0.7705
A15, mm	54 [49; 60]	50 [43; 55]	49 [47; 53]	49 [45; 54]	0.126	0.0635	0.9798	0.8012
A20, mm	57 [53; 61]	53 [47; 58]	52 [49; 56]	53 [49; 57.5]	0.1282	0.044	0.945	0.6919
A30, mm	58 [55; 63]	55 [49; 60]	55 [51; 57]	55 [51; 59]	0.1173	0.0467	0.9743	0.7143
ADP, AU×min	69 [42; 85]	57.5 [46.25; 66.5]	67 [54.5; 76.5]	63 [49.5; 71]	0.7853	0.9498	0.5866	0.2224
ASPI, AU×min	40 [21; 47]	26 [18; 40.75]	60 [45; 65]	58 [47.5; 68]	0.3568	0.0535	0.0001	0.0002
CFT, s	187 [138; 245]	195 [150; 277]	227 [155; 294]	222 [176; 275]	0.5056	0.2297	0.5663	0.6964
CLT, min	20.3 [15.7; 24.3]	20.5 [15.32; 26.02]	19.6 [16.92; 22.9]	23.2 [20.3; 30.4]	0.9157	0.9787	0.2036	0.9127
CS, mcm	1267.5 [1216; 1446]	1384.5 [1245.12; 1450]	1225 [1164.5; 1342]	1159 [1044.25; 1246.5]	0.6023	0.3568	0.0026	0.0988
CT, s	647 [503; 727]	722 [578; 844]	645 [542; 885]	717 [625; 834]	0.1715	0.562	0.6689	0.8297
D, a.u.	27381.5 [25505.75; 31961.5]	25461.5 [22630.75; 27474.5]	22996.5 [21665.75; 24478.25]	23565 [22216; 26854.5]	0.0792	0.0023	0.5056	0.0535
LI, %	44.5 [33.1; 49.9]	45.45 [31.82; 53.5]	44.6 [37.33; 50.38]	51 [47.4; 54.4]	0.8524	0.9157	0.1715	0.8587
LI60 (%)	95 [94; 97]	95 [93; 97]	95 [93; 97]	96 [93.5; 97]	0.4678	0.872	0.6059	0.6689
LOT, min	23.9 [20.8; 24.7]	23.4 [19.6; 28.12]	20.25 [18.38; 22.95]	23.8 [20.5; 27.5]	0.9669	0.3573	0.5663	0.4142
LP, %/min	5.2 [4; 7.4]	4.95 [3.3; 7.77]	5.15 [3.95; 6.53]	3.8 [3.1; 4.5]	0.8043	0.8012	0.1367	0.9437
LTE, min	22.7 [16.2; 28.9]	21.65 [16.68; 31.45]	22.75 [17.85; 29.55]	28.2 [24.6; 36.5]	1	0.8627	0.0732	0.8087
MCF, mm	59 [56; 64]	56 [50; 60]	56 [52; 58]	56 [52; 59]	0.0784	0.044	0.9265	0.9157
ML, %	20 [18; 25]	23 [21; 28]	23 [21; 26]	24 [19.5; 26]	0.1091	0.3532	0.3926	0.7189
Tlag, min	1.1 [1; 1.12]	1 [0.9; 1.17]	0.95 [0.8; 1]	1 [0.8; 1]	0.5964	0.0336	0.157	0.1361
TRAP-6, AU×min	106 [77; 123]	101.5 [82.25; 119]	97 [86; 106]	94 [86.5; 105]	0.9437	0.4976	0.3408	0.3483
Tsp, min	26.1 [22.65; 36.5]	31.7 [26.1; 53.2]	28.15 [21.4; 45.15]	43.7 [28; 51.75]	0.3408	0.817	0.7053	0.4829
V, μm/min	34.8 [31.28; 44.05]	34.75 [30.96; 38.68]	32.1 [30; 39.5]	29.2 [26; 32.9]	0.6644	0.3367	0.0075	0.5652
Vi, μm/min	58.7 [55.6; 65.3]	63.4 [58.5; 66.38]	56.1 [53.08; 57.77]	52.6 [48.95; 56.45]	0.5652	0.1361	0.0003	0.0027

Vst, μm/min	31.85 [31.17; 39.82]	34.7 [30.65; 38.6]	32.1 [30; 39.5]	29 [26; 32.71]	0.9217	0.7307	0.0069	0.6626
α, °	57 [49; 65]	55 [46.75; 63]	52 [45; 60]	52 [46; 57.5]	0.4288	0.1472	0.5536	0.4992

Green: *p* adj. < 0.1. Parameters of rotational thromboelastometry: CT - clotting time, CFT - clot formation time, A10-A30 - clot amplitudes at 10-30 min, MCF - maximum clot firmness, α - angle between the middle axis and the tangential line to the clotting curve through the 2-mm amplitude point, LI60 - clot lysis index at 60 min, ML - maximum lysis. Parameters of thrombodynamics: V - clot growth rate, Vi - initial clot growth rate, Vst – stationary clot growth rate, Tlag - Lag-time, the delay between the test start and the clot formation onset, CS - clot size, D - clot density, Tsp - spontaneous clots formation time, LOT - lysis onset time, LP - the rate of lysis progression, CLT - the clot lysis time, LI - percent of remaining clot density, LTE - the expected clot lysis time. Parameters of impedance aggregometry: ASPI - platelet activation with arachidonic acid, ADP- platelet activation with adenosine diphosphate, TRAP-6 - platelet activation with thrombin receptor-activated peptide-6.

Table S3. Functional groups of proteins analyzed in the study.

Group of proteins	Included proteins
Hemostasis	Alpha-2-antiplasmin, alpha-2-macroglobulin, beta-2-glycoprotein 1, C4b-binding protein alpha chain, coagulation factor IX, coagulation factor XII, coagulation factor XIII A chain, coagulation factor XIII B chain, fibrinogen beta chain, fibrinogen gamma chain, fibronectin, hyaluronan-binding protein 2, kallistatin, plasma serine protease inhibitor, protein Z-dependent protease inhibitor, prothrombin, serotransferrin, vitamin K-dependent protein S, vitronectin, carbonic anhydrase 1
Extracellular matrix	Proteins and regulators of extracellular matrix - fibronectin, tenascin-X, lumican, fibulin-1, vitronectin, alpha-1-antitrypsin, fetuin-B, inter-alpha-trypsin inhibitor heavy chain H2, tetranectin, Xaa-Pro dipeptidase, alpha-2-macroglobulin
Endothelium	Proteins regulating the state of endothelium - angiogenin, insulin-like growth factor-binding protein 2, insulin-like growth factor-binding protein 3, insulin-like growth factor-binding protein complex acid labile subunit, retinol-binding protein 4, pigment epithelium-derived factor, clusterin
Inflammation	Proteins involved in inflammation excluding complement system proteins - L-selectin, phosphatidylinositol-glycan-specific phospholipase D, plastin-2, attractin, ficolin-2, extracellular matrix protein 1, alpha-2-macroglobulin, fibrinogen beta chain, fibrinogen gamma chain, C-reactive protein, haptoglobin, leucine-rich alpha-2-glycoprotein, ceruloplasmin, hemopexin, lipopolysaccharide-binding protein
Complement system	Alpha-2-macroglobulin, C4b-binding protein alpha chain, complement C1q subcomponent subunit A, complement C1q subcomponent subunit B, complement C3, complement C4, complement C5, complement C6, complement C8 alpha chain, complement C8 beta chain, complement C9, complement factor B, complement factor I, ficolin-2, mannan-binding lectin serine protease 2A, plasma protease C1 inhibitor, vitronectin
Lipid metabolism	Apolipoprotein A-I, apolipoprotein A-IV, apolipoprotein B-100, apolipoprotein C-I, apolipoprotein C-II, apolipoprotein C-III, apolipoprotein C-IV, apolipoprotein E, apolipoprotein F, apolipoprotein L1,apolipoprotein M,

	hemopexin, phosphatidylcholine-sterol acyltransferase, phospholipid transfer protein, serum amyloid A-1 and A-2 proteins, zinc-alpha-2-glycoprotein
Calcification	Regulators of bone and vascular calcification - alpha-2-HS-glycoprotein, carbonic anhydrase 1, matrix Gla protein
Steroid hormone transport	Corticosteroid-binding globulin, sex hormone-binding globulin

Proteins were divided into the functional groups manually based on UniProt and NCBI gene databases, and articles in PubMed.

Table S4. Comparison of studied proteomics parameters between study groups.

Parameter	AMI control	AMI post-COVID	Control	Control post-COVID	*p* adj. AMI control vs AMI post-COVID	*p* adj. AMI control vs Control	*p* adj. AMI post-COVID vs Control post-COVID	*p* adj. AMI post-COVID vs Control
Alpha-1-antitrypsin	4850.4 [4328.2; 6422.7]	4603.1 [4255.75; 5162.05]	4477.9 [4143.35; 4858.5]	4241.5 [3836; 4652.25]	0.2451	0.126	0.0764	0.5799
Alpha-2-antiplasmin	404.52 [337.33; 431.16]	374.36 [327.72; 415.78]	377.82 [342.46; 410.59]	373.2 [347.68; 401.12]	0.3568	0.4526	0.9498	0.8584
Alpha-2-HS-glycoprotein	187.36 [134.2; 215.67]	178.36 [163.44; 197.78]	180.3 [165.81; 223.02]	188.91 [173.05; 211.91]	0.9265	0.6644	0.3563	0.5964
Alpha-2-macroglobulin	1120.2 [852.92; 1313.5]	929.15 [817.2; 1130.7]	918.36 [837.66; 1080.6]	1017.7 [908.24; 1159.85]	0.3568	0.3532	0.3576	0.9849
Angiogenin	8.5 [6.52; 14.68]	10.27 [6.64; 14.68]	5.62 [3.87; 8.53]	4.78 [3.52; 7.29]	0.9265	0.024	0.0001	0.0036
Apolipoprotein A-I	15805 [6799.8; 16384]	15607 [8164; 18507]	20361 [16404.5; 22429]	20211 [18708.5; 23508.5]	0.5493	0.0056	0.0002	0.0075
Apolipoprotein A-IV	76.38 [59.78; 105.86]	85.54 [67.3; 104.11]	86.77 [75.24; 114.92]	95.95 [85.2; 108.26]	0.817	0.3408	0.0792	0.4291
Apolipoprotein B-100	32.01 [27.79; 38.29]	32.56 [25.23; 42.47]	29.79 [25.4; 33.93]	24.97 [19.95; 29.88]	0.8867	0.5731	0.029	0.367
Apolipoprotein C-I	450.5 [383.4; 697.52]	616.32 [420.86; 827.77]	634.85 [507.28; 712.32]	651.92 [565.49; 724.19]	0.3408	0.154	0.7145	0.9743
Apolipoprotein C-II	77.56 [66.17; 91.83]	83.13 [53.63; 108.04]	55.07 [43.06; 87.77]	60.02 [47.87; 70.24]	0.8933	0.1367	0.0891	0.0764
Apolipoprotein C-III	393.85 [314.52; 438.92]	356.49 [270.98; 482.95]	290.97 [228.26; 382.9]	274.6 [222.4; 334.56]	0.817	0.0792	0.0134	0.1028
Apolipoprotein C-IV	21.44 [12.82; 28.16]	20.3 [16.09; 35.61]	18.59 [12.36; 25.52]	16.72 [12.34; 28.02]	0.679	0.6562	0.2497	0.3516
Apolipoprotein E	123.35 [105.36; 159]	149.54 [130.06; 180.2]	146.62 [119.59; 173.57]	141.03 [127.72; 158.73]	0.3522	0.3396	0.7699	0.943
Apolipoprotein F	152.14 [102.22; 187.36]	128.85 [110.41; 161.37]	148.31 [134.47; 180.4]	143.44 [131.08; 162.45]	0.7109	0.6689	0.336	0.0764
Apolipoprotein L1	44.29 [42.08; 53.86]	54.58 [47.8; 69.57]	49.42 [43.78; 63.75]	51.45 [44.65; 59.09]	0.1028	0.3997	0.336	0.3568
Apolipoprotein M	79.58 [57.2; 114.06]	93.09 [69.78; 118.72]	75.13 [67.38; 100.5]	79.64 [67.43; 95.54]	0.6676	0.9787	0.3757	0.3576
Attractin	34.53 [29.33; 38.11]	36.53 [32.11; 40.24]	33.07 [29.08; 37.77]	34.97 [31.82; 38.33]	0.4293	0.5944	0.5056	0.0764
Beta-2-glycoprotein 1	295.42 [233.94; 323.89]	261.99 [241.12; 295.69]	233.29 [215.05; 270.67]	245.42 [220.39; 265.85]	0.5592	0.057	0.1008	0.0732

C-reactive protein	4.5 [2.23; 10.9]	3.73 [2.33; 6.91]	1.52 [0.93; 2.44]	2.12 [1.42; 4.27]	0.3997	0.0026	0.0666	0.0022
C4b-binding protein alpha chain	255.69 [224.54; 357.26]	215.84 [176.55; 284.52]	221 [164.42; 276.26]	295.53 [234.54; 329.51]	0.0811	0.1282	0.0187	0.9552
Carbonic anhydrase 1	3.07 [1.69; 5.14]	2.38 [1.35; 3.74]	1.81 [1.38; 3.59]	1.69 [1.28; 2.71]	0.4895	0.4526	0.3532	0.7853
Ceruloplasmin	117.91 [99.87; 153.04]	109.53 [93.48; 141.34]	105.34 [92.19; 126.28]	107.57 [96.29; 127.71]	0.5364	0.3568	0.9798	0.6975
Clusterin	5946.3 [5104.3; 6786.1]	5750.3 [5027.4; 6941.15]	6314 [5596.5; 7532.65]	6154.5 [5333.6; 6911.35]	0.6689	0.5652	0.4473	0.1884
Coagulation factor IX	13.34 [11.47; 14.87]	12.07 [10.37; 13.33]	10.65 [8.79; 12.12]	10.4 [9; 12.49]	0.2463	0.0053	0.1326	0.0754
Coagulation factor XII	20.17 [17.8; 21.55]	15.62 [13.72; 20.66]	22.11 [17.1; 25.3]	19.74 [17.39; 24.29]	0.1326	0.3568	0.044	0.0134
Coagulation factor XIII A chain	5.61 [4.22; 6.83]	6.2 [5.11; 7.51]	6.01 [5.23; 7]	6.13 [5.37; 7.09]	0.4437	0.6689	0.8933	0.693
Coagulation factor XIII B chain	12 [7.75; 14.65]	9.47 [7.55; 11.72]	8.8 [5.62; 11.52]	7.92 [6.26; 9.83]	0.5955	0.1698	0.0364	0.3568
Complement C1q subcomponent subunit A	21.45 [16.74; 24.95]	23.05 [17.97; 25.26]	19.54 [17.88; 21.72]	17.68 [16.52; 20.72]	0.6975	0.4894	0.024	0.126
Complement C1q subcomponent subunit B	18.68 [18; 20.7]	17.73 [15.33; 19.71]	18.24 [14.15; 21]	16.85 [15.05; 18.93]	0.3408	0.3926	0.4286	0.9437
Complement C1s subcomponent	421.74 [374.21; 490.98]	409.16 [352.74; 442.65]	402.04 [371.22; 441.98]	378.19 [359.47; 429]	0.3538	0.3997	0.7934	0.8933
Complement C3	485.03 [439.91; 527.76]	444.43 [409.06; 526.9]	416.34 [362.92; 492.3]	400.98 [375.48; 508.1]	0.4993	0.0411	0.158	0.1091
Complement C4	459.97 [4.11; 527.85]	340.07 [4.28; 464.46]	418.35 [286.36; 531.12]	435.49 [365.32; 520.32]	0.6626	0.8297	0.0635	0.336
Complement C5	55.16 [45.59; 69.41]	52.34 [48.87; 59.33]	42.52 [39.23; 46.65]	42.78 [39.4; 46.85]	0.4678	0.0001	0.0002	0
Complement C6	20.09 [12.86; 25.19]	21.81 [14.68; 30.44]	16.99 [12.29; 19.95]	12.77 [9.91; 19.58]	0.4993	0.3568	0.0049	0.044
Complement C8 alpha chain	41.96 [36.06; 45.94]	37.39 [33.77; 42.26]	38.64 [33.75; 44.15]	41.32 [38.23; 44.63]	0.2284	0.4437	0.2451	0.6902
Complement C8 beta chain	7.14 [5.82; 8.36]	6.85 [5.55; 7.86]	5.87 [5.43; 7.68]	7.31 [5.31; 8.83]	0.6975	0.3408	0.7116	0.4163
Complement C9	21.47 [19.34; 31.72]	22.48 [17.38; 25.29]	17.95 [15.45; 20.92]	18.19 [15.98; 22.26]	0.693	0.0764	0.0792	0.024
Complement factor B	111.12 [98; 121.92]	98.92 [87.6; 113.9]	87.76 [71.42; 95.13]	89.64 [76.21; 100.85]	0.1971	0.0049	0.0811	0.0364
Complement factor I	37.55 [34.34; 39.47]	34.6 [31.17; 38.1]	29.84 [26.64; 33.96]	32.77 [29.37; 36.07]	0.1361	0.0015	0.386	0.0467
Corticosteroid-binding globulin	572.44 [504.68; 675.83]	521.88 [482.4; 609.12]	600.08 [573.52; 645.88]	616.34 [563.62; 664.28]	0.1971	0.4976	0.0026	0.0075

Extracellular matrix protein 1	9.05 [6.36; 10.38]	10.04 [8.75; 11.22]	10.93 [8.72; 11.74]	9.14 [8.1; 10.03]	0.2224	0.158	0.2198	0.6012
Fetuin-B	4.75 [4.03; 5.92]	4.84 [3.93; 5.29]	5.17 [3.74; 5.73]	4.28 [3.6; 4.9]	0.5408	0.9299	0.336	0.4976
Fibrinogen beta chain	3127.4 [2645.7; 4013.6]	2664.4 [2237.15; 3157.8]	2269.1 [1940.45; 2495.35]	2303.2 [2083.05; 2711.95]	0.0732	0.0022	0.0784	0.0187
Fibrinogen gamma chain	2287.4 [2035.9; 2974.8]	2090.4 [1823.7; 2309.3]	1823.5 [1662.05; 1979.1]	1773.4 [1650.7; 2018.25]	0.1851	0.0032	0.0065	0.0134
Fibronectin	165.02 [84.22; 221.98]	149.12 [113.07; 169.29]	137.1 [105.21; 156.99]	120.91 [95.54; 144.49]	0.7109	0.5408	0.0891	0.5056
Fibulin-1	19.73 [15.31; 23.49]	17.29 [14.55; 20.74]	18.41 [16.52; 22.8]	18.67 [17.23; 20.68]	0.3997	0.9627	0.3006	0.3408
Ficolin-2	2.77 [2.08; 4.65]	3.76 [2.47; 5.95]	2.81 [1.63; 4.32]	2.32 [1.92; 3.65]	0.4829	0.6689	0.1091	0.2108
Haptoglobin	4013.4 [2694.1; 6977.3]	3530.1 [2560.6; 4426.1]	2925.2 [1939.8; 3498.8]	3358.5 [2611.7; 4088.9]	0.3265	0.029	0.5592	0.0467
Hemopexin	1493 [1316; 1630.9]	1370.4 [1261.2; 1469.45]	1363.6 [1238.35; 1462]	1373.1 [1255.35; 1482.25]	0.2094	0.1653	0.9437	0.8071
Hyaluronan-binding protein 2	12.11 [11.05; 13.73]	11.26 [9.97; 12.94]	11.73 [11.03; 12.33]	11.28 [10.46; 12.22]	0.1662	0.4976	0.5652	0.4811
Insulin-like growth factor-binding protein 2	1.44 [0.72; 3.08]	1.09 [0.64; 2]	0.85 [0.38; 1.96]	0.95 [0.79; 1.47]	0.3435	0.1367	0.6911	0.558
Insulin-like growth factor-binding protein 3	12.94 [10.24; 15.09]	12.72 [10.44; 14.25]	13.92 [12.47; 16.3]	13.69 [12.56; 15.33]	0.9626	0.2439	0.2451	0.1091
Insulin-like growth factor-binding protein complex acid labile subunit	11.28 [8.37; 13.99]	11.53 [8.68; 12.68]	13.61 [11.45; 15.09]	12.69 [10.7; 13.93]	0.7699	0.0754	0.0811	0.0049
Inter-alpha-trypsin inhibitor heavy chain H2	70.3 [65.68; 83.53]	74.99 [63.55; 82.29]	70.23 [63.76; 84.03]	66.98 [63.41; 73.63]	0.8509	0.9451	0.2934	0.9798
Kallistatin	8.52 [6.76; 9.24]	8.58 [7.39; 9.68]	8.14 [7.39; 9.1]	8.02 [7.11; 9.55]	0.8404	0.7106	0.7523	0.6059
L-selectin	59.2 [45.48; 71.91]	59.76 [50.53; 71.96]	68.63 [54.63; 76.12]	68.67 [60.57; 78.12]	0.9743	0.5731	0.0364	0.3516
Leucine-rich alpha-2-glycoprotein	50.51 [40.16; 83.39]	44.7 [38.61; 53.15]	42.02 [37.36; 46.21]	40.85 [35.4; 48.74]	0.2925	0.0608	0.3576	0.2184
Lipopolysaccharide-binding protein	28.19 [20.41; 35.83]	23.03 [17.87; 26.87]	17.44 [13.62; 21.21]	20.43 [16.62; 23.31]	0.0635	0.0009	0.208	0.0075
Lumican	17.4 [15.21; 19.6]	17.44 [15.99; 20.77]	15.03 [12.94; 17.44]	16.78 [15.47; 18.41]	0.7339	0.208	0.3532	0.0071
Mannan-binding lectin serine protease 2A	89.92 [66.11; 115.42]	72.63 [54.62; 90.38]	69.06 [53.08; 84.64]	42.89 [32.93; 71.61]	0.1732	0.1282	0.0134	0.7123
Matrix Gla protein	2.18 [1.27; 2.63]	1.8 [1.24; 2.2]	1.2 [0.89; 1.6]	1.38 [1.15; 1.76]	0.4437	0.0364	0.0869	0.0509
Phosphatidylcholine-sterol acyltransferase	30.82 [24.91; 36.09]	27.41 [22.01; 31.95]	30.58 [26.22; 35.09]	29.6 [25.82; 35.54]	0.2966	0.9787	0.3568	0.168

Phosphatidylinositol-glycan-specific phospholipase D	28.71 [26.24; 31.57]	29.92 [25.8; 33.98]	26.62 [23.76; 30.99]	25.45 [21.66; 29.12]	0.9157	0.3532	0.0336	0.1662
Phospholipid transfer protein	4.26 [3.7; 4.55]	4.57 [3.43; 5.43]	4.57 [4.06; 4.89]	4.67 [4.17; 5.57]	0.5652	0.367	0.5195	0.9092
Pigment epithelium-derived factor	19.03 [17.4; 21.19]	15.77 [13.75; 18.26]	13.54 [12.01; 16.97]	16.32 [12.47; 17.79]	0.0666	0.0026	0.4728	0.0535
Plasma protease C1 inhibitor	166.85 [147.09; 220.02]	142.37 [131.48; 167.3]	139.93 [126.64; 160.72]	155.36 [134.32; 174.58]	0.0187	0.0075	0.5272	0.5932
Plasma serine protease inhibitor	65.91 [55.94; 69.3]	58.4 [52.61; 67.26]	62.04 [51.73; 66.95]	55.29 [46.22; 64.3]	0.5652	0.7699	0.1884	0.9437
Plastin-2	6.94 [5.84; 7.85]	7.02 [5.81; 7.86]	6.93 [5.7; 7.38]	6.72 [5.88; 7.28]	1	0.6927	0.7934	0.5932
Protein Z-dependent protease inhibitor	38.46 [29.31; 43.04]	32.97 [27.24; 37.58]	34.45 [29.99; 40.8]	35.57 [31.21; 39.8]	0.3408	0.6562	0.3006	0.5989
Prothrombin	1026.8 [836.9; 1217.4]	987.23 [820.16; 1122.35]	968.31 [858.76; 1017.1]	970.29 [907.05; 1057.65]	0.6626	0.433	0.9498	0.5465
Retinol-binding protein 4	112.33 [78.45; 158.65]	94.19 [76.4; 115.14]	85.46 [79.26; 100.2]	104.7 [80.56; 127.97]	0.1971	0.195	0.4959	0.5652
Serotransferrin	1421.3 [1384.9; 1564.5]	1405.8 [1274.65; 1636.65]	1521.9 [1383.8; 1989]	1419.2 [1322.45; 1581.5]	0.5811	0.3753	0.8038	0.1054
Serum amyloid A-1 and A-2 proteins	13.41 [3.94; 42.99]	7.84 [4.03; 11.76]	3.82 [2.59; 7.08]	4.85 [2.84; 7.54]	0.3576	0.0792	0.1116	0.1282
Sex hormone-binding globulin	15.64 [12.56; 19.38]	16.89 [12.7; 20.68]	19.96 [17.41; 35.63]	25.45 [19.24; 35.67]	0.7859	0.0187	0.004	0.0075
Tenascin-X	36.88 [28.12; 48.78]	30.94 [26.06; 38.34]	25.4 [21.6; 32.52]	23.52 [20.07; 29.56]	0.3568	0.0364	0.0046	0.0754
Tetranectin	242.98 [222.38; 275.64]	265.08 [231.85; 313.45]	284.27 [232.3; 314.18]	317.13 [274.67; 367.06]	0.4087	0.36	0.0075	0.8012
Vitamin K-dependent protein S	35.43 [31.16; 40.88]	32.16 [29.34; 34.61]	31.75 [26.62; 33.99]	30.82 [29.72; 32.38]	0.0535	0.0187	0.3712	0.367
Vitronectin	571 [535.01; 646.37]	570.47 [481.48; 621.57]	518.36 [438.74; 558.46]	488.5 [437.46; 577.18]	0.6012	0.0187	0.157	0.119
Xaa-Pro dipeptidase	1.1 [0.88; 1.4]	1 [0.9; 1.17]	1.02 [0.9; 1.2]	0.99 [0.86; 1.21]	0.5465	0.7923	0.8627	0.9669
Zinc-alpha-2-glycoprotein	25.48 [20.53; 31.23]	21.12 [19.11; 25.84]	20.35 [18.09; 26.86]	26.24 [22.36; 30.01]	0.157	0.2439	0.0467	0.7783

Green: *p* adj. < 0.1

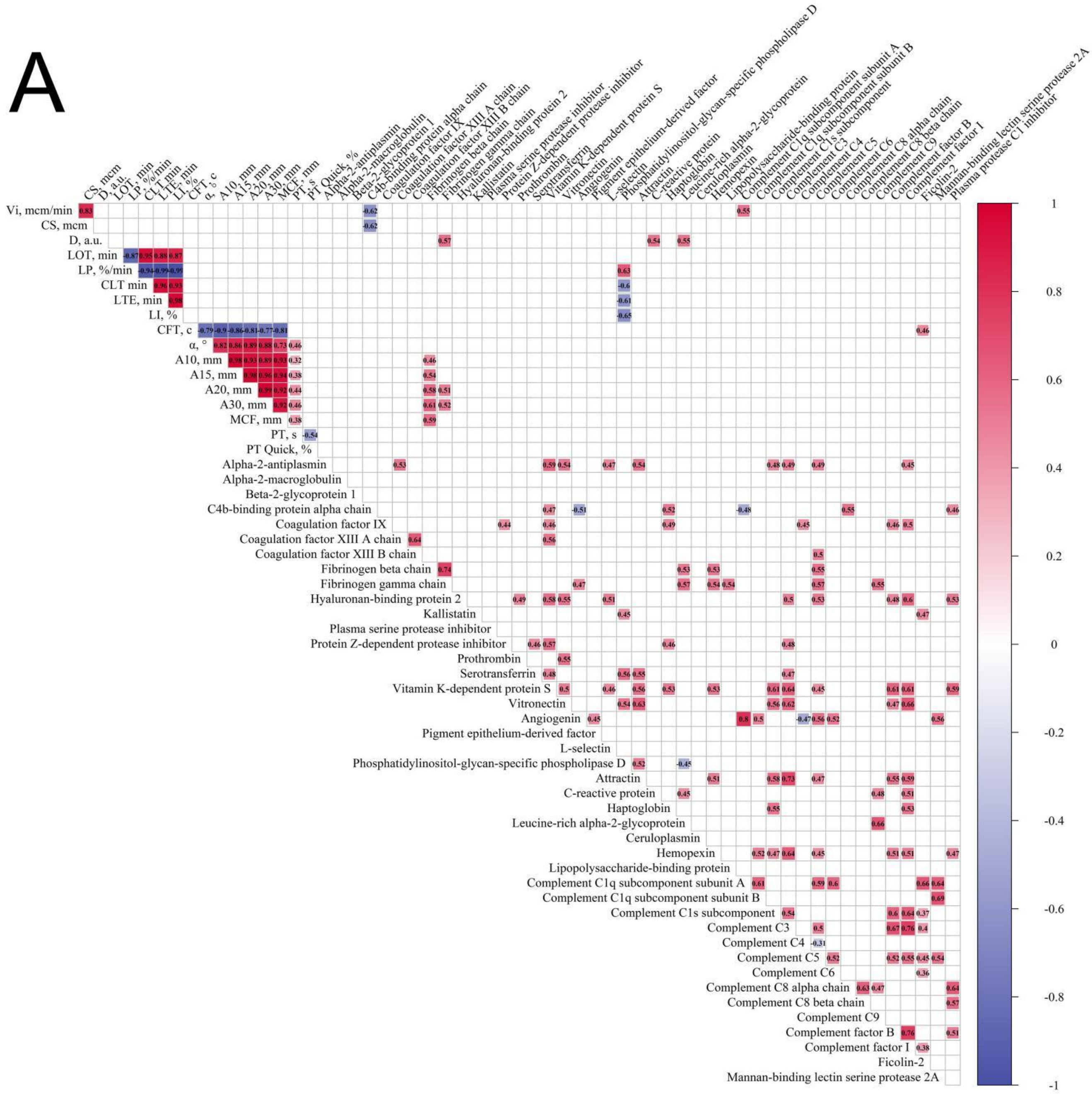
A
CS, mcm
D, a.u.
LOT, min
LP, %/min
CLT min
LTE, min
LI, %
CFT, c
α, °
A10, mm
A15, mm
A20, mm
A30, mm
MCF, mm
PT, s
PT Quick, %
Alpha-2-antiplasmin
Alpha-2-macroglobulin
Beta-2-glycoprotein 1
C4b-binding protein alpha chain
Coagulation factor IX
Coagulation factor XIII A chain
Coagulation factor XIII B chain
Fibrinogen beta chain
Fibrinogen gamma chain
Hyaluronan-binding protein 2
Kallistatin
Plasma serine protease inhibitor
Protein Z-dependent protease inhibitor
Prothrombin
Serotransferrin
Vitamin K-dependent protein S
Vitronectin
Angiogenin
Pigment epithelium-derived factor
L-selectin
Phosphatidylinositol-glycan-specific phospholipase D
Attractin
C-reactive protein
Haptoglobin
Leucine-rich alpha-2-glycoprotein
Ceruloplasmin
Hemopexin
Lipopolysaccharide-binding protein
Complement C1q subcomponent subunit A
Complement C1q subcomponent subunit B
Complement C1s subcomponent
Complement C3
Complement C4
Complement C5
Complement C6
Complement C8 alpha chain
Complement C8 beta chain
Complement C9
Complement factor B
Complement factor I
Ficolin-2
Mannan-binding lectin serine protease 2A
Plasma protease C1 inhibitor
Vi, mcm/min
1
0.8
0.6
0.4
0.2
0
-0.2
-0.4
-0.6
-0.8
-1

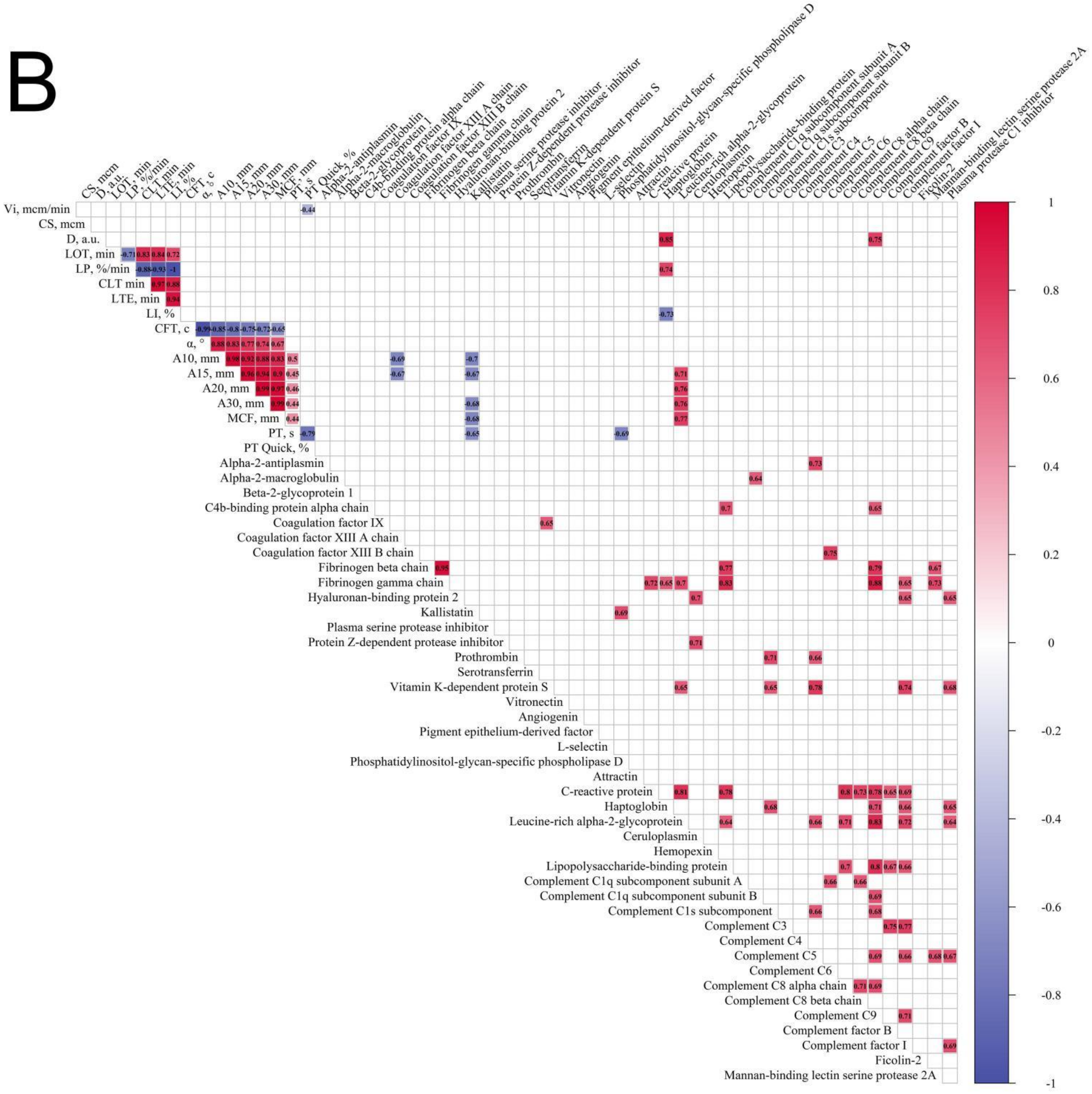
B
Vi, mcm/min
CS, mcm
D, a.u.
LOT, min
LP, %/min
CLT min
LTE, min
LI, %
CFT, c
α, °
A10, mm
A15, mm
A20, mm
A30, mm
MCF, mm
PT, s
PT Quick, %
Alpha-2-antiplasmin
Alpha-2-macroglobulin
Beta-2-glycoprotein 1
C4b-binding protein alpha chain
Coagulation factor IX
Coagulation factor XIII A chain
Coagulation factor XIII B chain
Fibrinogen beta chain
Fibrinogen gamma chain
Hyaluronan-binding protein 2
Kallistatin
Plasma serine protease inhibitor
Protein Z-dependent protease inhibitor
Prothrombin
Serotransferrin
Vitamin K-dependent protein S
Vitronectin
Angiogenin
Pigment epithelium-derived factor
L-selectin
Phosphatidylinositol-glycan-specific phospholipase D
Attractin
C-reactive protein
Haptoglobin
Leucine-rich alpha-2-glycoprotein
Ceruloplasmin
Hemopexin
Lipopolysaccharide-binding protein
Complement C1q subcomponent subunit A
Complement C1q subcomponent subunit B
Complement C1s subcomponent
Complement C3
Complement C4
Complement C5
Complement C6
Complement C8 alpha chain
Complement C8 beta chain
Complement C9
Complement factor B
Complement factor I
Ficolin-2
Mannan-binding lectin serine protease 2A
Plasma protease C1 inhibitor
1
0.8
0.6
0.4
0.2
0
-0.2
-0.4
-0.6
-0.8
-1

C

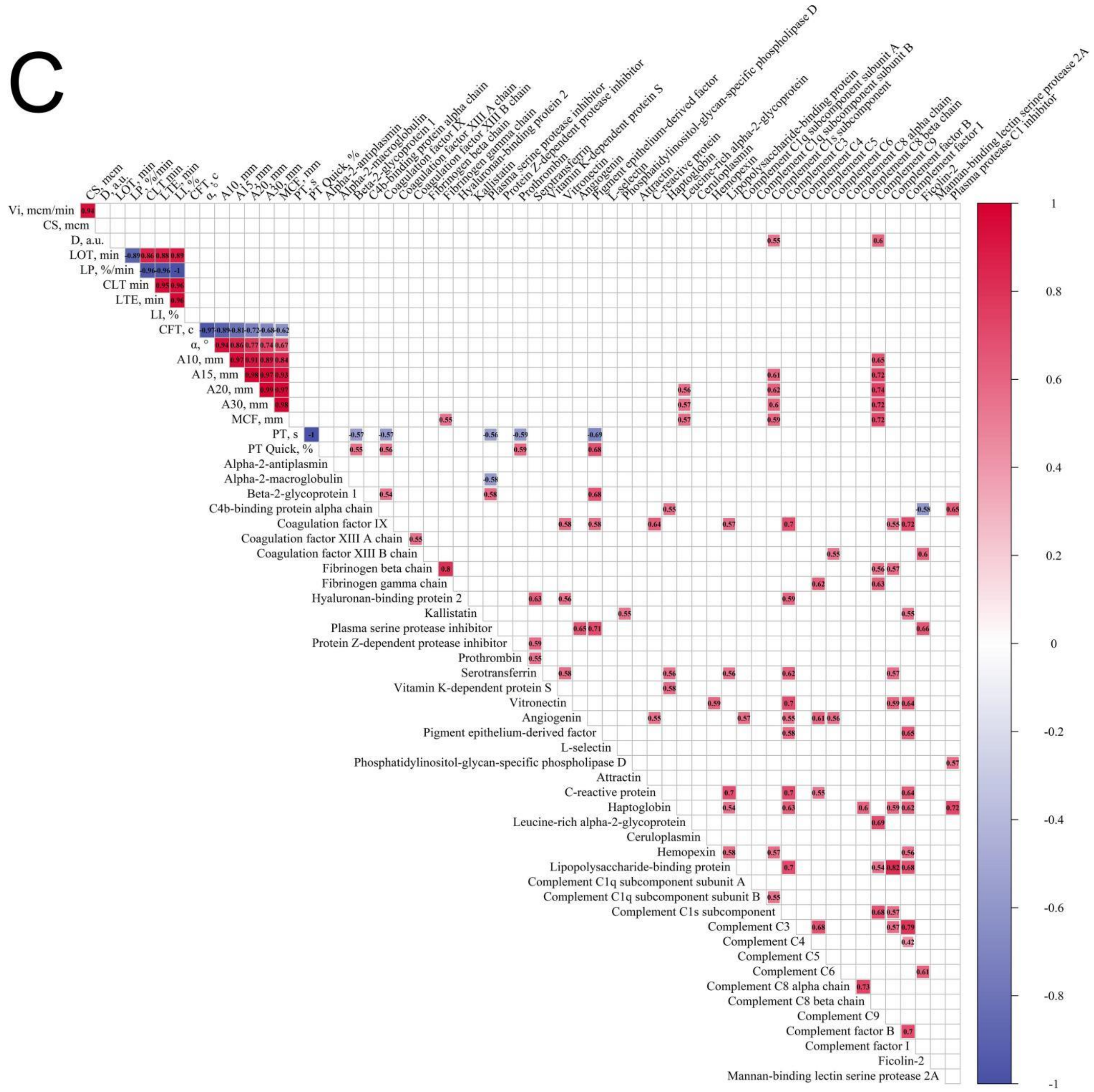
Vi, mcm/min
CS, mcm
D, a.u.
LOT, min
LP, %/min
CLT min
LTE, min
LI, %
CFT, c
α, °
A10, mm
A15, mm
A20, mm
A30, mm
MCF, mm
PT, s
PT Quick, %
Alpha-2-antiplasmin
Alpha-2-macroglobulin
Beta-2-glycoprotein 1
C4b-binding protein alpha chain
Coagulation factor IX
Coagulation factor XIII A chain
Coagulation factor XIII B chain
Fibrinogen beta chain
Fibrinogen gamma chain
Hyaluronan-binding protein 2
Kallistatin
Plasma serine protease inhibitor
Protein Z-dependent protease inhibitor
Prothrombin
Serotransferrin
Vitamin K-dependent protein S
Vitronectin
Angiogenin
Pigment epithelium-derived factor
L-selectin
Phosphatidylinositol-glycan-specific phospholipase D
Attractin
C-reactive protein
Haptoglobin
Leucine-rich alpha-2-glycoprotein
Ceruloplasmin
Hemopexin
Lipopolysaccharide-binding protein
Complement C1q subcomponent subunit A
Complement C1q subcomponent subunit B
Complement C1s subcomponent
Complement C3
Complement C4
Complement C5
Complement C6
Complement C8 alpha chain
Complement C8 beta chain
Complement C9
Complement factor B
Complement factor I
Ficolin-2
Mannan-binding lectin serine protease 2A
Plasma protease C1 inhibitor
1
0.8
0.6
0.4
0.2
0
-0.2
-0.4
-0.6
-0.8
-1

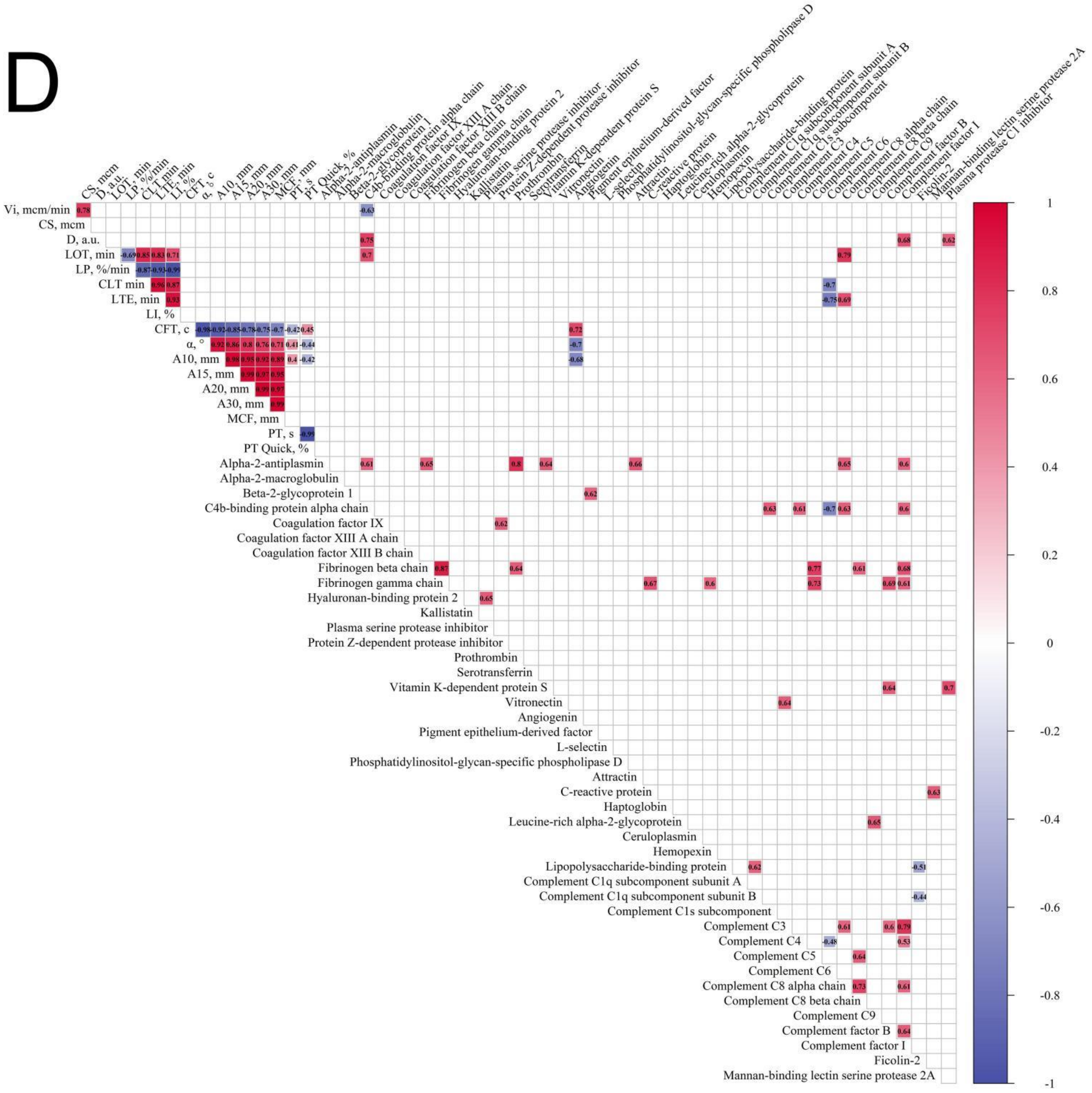

Figure S4. Main correlations between proteomics data and parameters of hemostasis. Targeted proteomic analysis was carried out using liquid chromatography-tandem mass spectrometry (LC-MS/MS) with multiple reaction monitoring (MRM). Parameters of hemostasis include results of rotational thromboelastometry, thrombodynamics, and coagulation blood tests. Correlation matrices demonstrate only those parameters of hemostasis and proteins connected to hemostasis which correlated with proteins connected to inflammation (our division of proteins into functional groups is presented in Table S3) and components of the complement system in at least one study group. Also, we included proteins connected to inflammation and components of the complement system if they correlated with parameters of hemostasis and proteins connected to hemostasis in at least one study group. The full correlation tables are presented in Tables S6 (AMI post-COVID), S7 (AMI control), S8 (control post-COVID), S9 (control). Full correlation tables include all the 81 proteins and all the parameters of hemostasis analyzed in the study, and p adjustment was done for this list of correlations.

Correlations with p adj. < 0.05 and Spearman's correlation coefficient > 0.3 are shown. Red – positive correlations, blue – negative correlations. Parameters of coagulation blood tests: PT - prothrombin time (sec), PT Quick – Quick prothrombin time test (%). Parameters of rotational thromboelastometry: CFT - clot formation time (sec), A10-A30 - clot amplitudes at 10-30 min (mm), MCF - maximum clot firmness (mm), α - angle between the middle axis and the tangential line to the clotting curve through the 2-mm amplitude point (°). Parameters of thrombodynamics: CS - clot size (μm), D - clot density (arb units), LOT - lysis onset time (min), LP - the rate of lysis progression (%/min), CLT - the clot lysis time (min), LI - percent of remaining clot density (%), LTE - the expected clot lysis time (min).
(**A**) AMI post-COVID group; (**B**) AMI control group; (**C**) control post-COVID group; (**D**) control group.

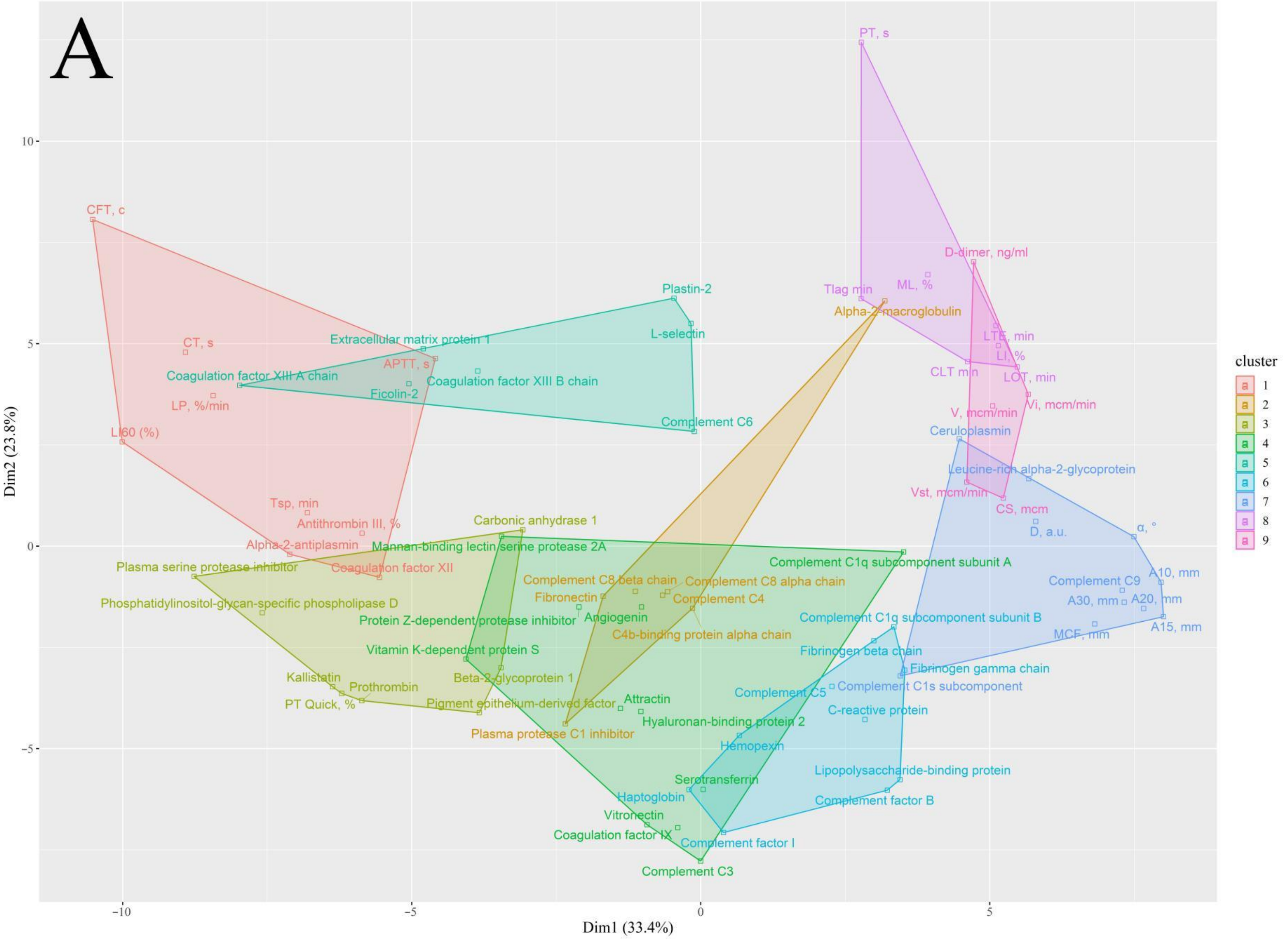
A
PT, s
D-dimer, ng/ml
Tlag min
ML, %
Alpha-2-macroglobulin
LTE, min
LI, %
CLT min
LOT, min
V, mcm/min
Vi, mcm/min
Ceruloplasmin
Leucine-rich alpha-2-glycoprotein
Vst, mcm/min
CS, mcm
D, a.u.
α, °
Complement C9
A10, mm
A30, mm
A20, mm
MCF, mm
A15, mm
CFT, c
CT, s
Extracellular matrix protein 1
APTT, s
Coagulation factor XIII A chain
Coagulation factor XIII B chain
Ficolin-2
LP, %/min
LI60 (%)
Plastin-2
L-selectin
Complement C6
Tsp, min
Antithrombin III, %
Alpha-2-antiplasmin
Coagulation factor XII
Carbonic anhydrase 1
Mannan-binding lectin serine protease 2A
Plasma serine protease inhibitor
Phosphatidylinositol-glycan-specific phospholipase D
Complement C8 beta chain
Complement C8 alpha chain
Fibronectin
Complement C4
Angiogenin
Protein Z-dependent protease inhibitor
C4b-binding protein alpha chain
Complement C1q subcomponent subunit A
Complement C1q subcomponent subunit B
Vitamin K-dependent protein S
Fibrinogen beta chain
Fibrinogen gamma chain
Kallistatin
Prothrombin
Beta-2-glycoprotein 1
Complement C1s subcomponent
PT Quick, %
Pigment epithelium-derived factor
Attractin
Complement C5
C-reactive protein
Hyaluronan-binding protein 2
Plasma protease C1 inhibitor
Hemopexin
Lipopolysaccharide-binding protein
Serotransferrin
Haptoglobin
Complement factor B
Vitronectin
Coagulation factor IX
Complement factor I
Complement C3
10
5
0
−5
−10
−5
0
5
Dim2 (23.8%)
Dim1 (33.4%)
cluster
1
2
3
4
5
6
7
8
9

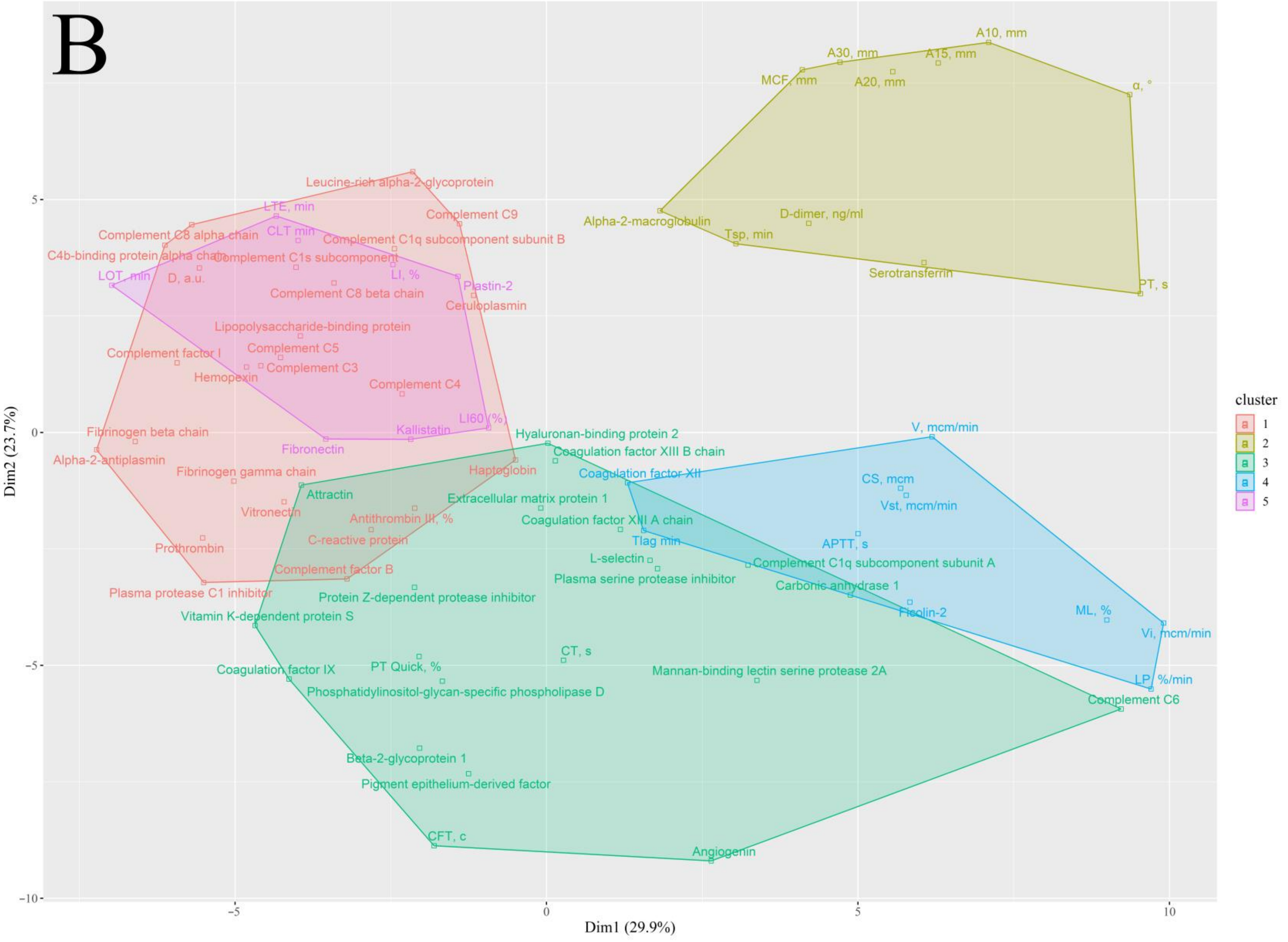

Figure S5. Clusterization of parameters of hemostasis, and proteins involved in inflammation and hemostasis, components of the complement system, and two proteins affecting endothelium (angiogenin, PEDF). We performed clusterization using the k-medoids algorithm and a correlation

matrix used as a matrix of distance. For defining the number of clusters, we used gap-statistics via bootstrapping with Monte-Carlo simulation and different centroids. Parameters of coagulation blood tests: APTT - activated partial thromboplastin time (sec), PT - prothrombin time (sec), PT Quick – Quick prothrombin time test (%). Parameters of rotational thromboelastometry: CT - clotting time (sec), CFT - clot formation time (sec), A10-A30 - clot amplitudes at 10-30 min (mm), MCF - maximum clot firmness (mm), α - angle between the middle axis and the tangential line to the clotting curve through the 2-mm amplitude point (°), LI60 - clot lysis index at 60 min (%), ML - maximum lysis (%). Parameters of thrombodynamics: V - clot growth rate (μm /min), Vi - initial clot growth rate (μm /min), Vst – stationary clot growth rate (μm/min), Tlag - Lag-time, the delay between the test start and the clot formation onset (min), CS - clot size (μm), D - clot density (arb units), Tsp - spontaneous clots formation time (min), LOT - lysis onset time (min), LP - the rate of lysis progression (%/min), CLT - the clot lysis time (min), LI - percent of remaining clot density (%), LTE - the expected clot lysis time (min).
(**A**) control post-COVID group; (**B**) control group.

Validation of proteomics results for C-reactive protein and fibrinogen beta chain

To validate the results of targeted proteomics we measured C-reactive protein and fibrinogen with other methods.

Blood was collected in S-Monovette 7,5 ml Z-Gel (REF 01.1602.001) tubes for C-reactive protein and S-Monovette 5 ml 9NC (REF 05.10I 71.001) tubes for fibrinogen measurement. C-reactive protein (CRP) was measured on Siemens ADVIA 2400 Chemistry Analyzer (Siemens Healthcare Diagnostics Inc, USA) with ADVIA® Chemistry Wide Range C-Reactive Protein Reagents (REF 829585). Clauss fibrinogen was automatically calculated after measurement on ACL TOP 300 CTS (Instrumentation Laboratory, USA) with HemosIL Fibrinogen-C XL (REF 00020003900).

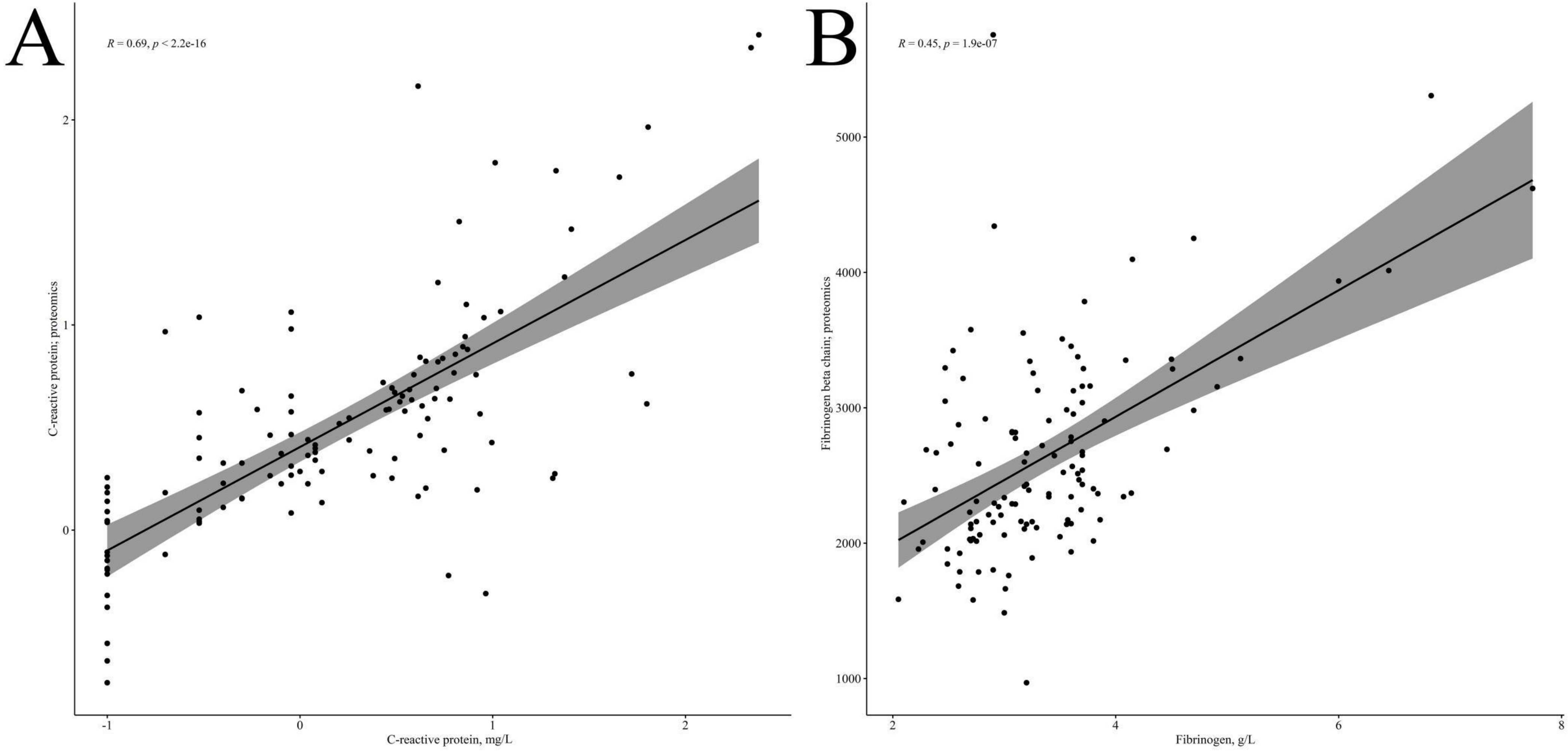

Figure S6. Validation of the results of targeted proteomics measured by liquid chromatography-tandem mass spectrometry (LC-MS/MS) with multiple reaction monitoring (MRM) with other methods. (**A**) correlation of C-reactive protein measured by proteomics (Y axis) with C-reactive protein measured on Siemens ADVIA 2400 Chemistry Analyzer, Spearman's correlation coefficient = 0.69, $p < 0.05$; (**B**) correlation of fibrinogen beta chain measured by proteomics with Clauss fibrinogen measured on ACL TOP 300 CTS, Spearman's correlation coefficient = 0.45, $p < 0.05$.
